# German college students’ mental health state and their willingness to use mental health prevention: An online survey during the COVID-19 pandemic

**DOI:** 10.1016/j.heliyon.2025.e42290

**Published:** 2025-01-31

**Authors:** Lena Ehl, Christin Scheiner, Antonia Wasserscheid, Grit Hein, Matthias Gamer, Arne Bürger

**Affiliations:** a Department of Child and Adolescent Psychiatry, Psychosomatics and Psychotherapy, Center of Mental Health, University Hospital of Würzburg, Germany; b German Center of Prevention Research in Mental Health, University of Würzburg, Würzburg, Germany; c Translational Social Neuroscience Unit, Department of Psychiatry, Psychosomatic and Psychotherapy, University Hospital Würzburg, Würzburg, Germany; d Department of Psychology, University of Würzburg, Würzburg, Germany

**Keywords:** Mental health, Universal prevention, Indicated prevention, Mood disorders, Anxiety disorders, College students

## Abstract

The number of college students suffering from mental illnesses has been rising for several years. The COVID-19 pandemic has disproportionately affected young adults. Mental health prevention is essential in order to effectively reduce the incidence of mental disorders and may help to counteract chronic mental disorders in the long term. Data were derived from a German online survey of 1334 college students (*M*_AGE_ = 24.75, *SD*_AGE_ = 3.32, [19–42] years) conducted in autumn 2021. Besides validated questionnaires (PHQ-2, ASI-3, CD-RISC-10) to assess their mental health status, we asked specific questions on students’ general interest in mental health prevention, whether students knew where to get help, and how many activities they engaged in to maintain their mental health. Students' overall interest in mental health prevention was high. Participants with clinically relevant scores were significantly more interested in prevention offers compared to those with clinically normal scores. Females engaged in significantly more activities to strengthen their mental health during the pandemic, and showed significantly higher anxiety scores and significantly lower resilience scores compared to their male counterparts. According to our results, students show decreasing mental health and resilience in times of crisis. Overall, motivation to seek professional support is high but knowledge about where to find such support is low. Based on these results, we conclude that easily accessible and low-threshold mental health prevention offers should be integrated into university curricula.

## Highlights

• Many students report depressive and anxiety symptoms, along with low resilience.
• Students, especially those with mental health issues, show high interest in mental health prevention.
• Interventions should be short, accessible, and combine online and face-to-face services.
• Crisis and advice contact options should be more prominent.

## 1 Introduction

Late adolescence and early/emerging adulthood are considered as particularly vulnerable life stages, with the college years being an especially crucial period [1]. Indeed, a fifth of college students meet the diagnostic criteria for a mental disorder, with anxiety disorders and depression being the two most common disorders among this group [2,3]. Reported prevalence rates for anxiety disorders range from 11 % to 17 %, while rates for depression range from 6 % to 14 % [2,[4], [5], [6]]. Even before the outbreak of the COVID-19 pandemic, experts had already identified an increase in mental disorders among college students [3,7].

The COVID-19 pandemic is disproportionately affecting young adults [8], with college students facing significant academic changes and disruptions [9]. For some, the disruptions have even meant delaying graduation and becoming less competitive on the job market [10]. Furthermore, student status was found to be significantly associated with higher levels of stress, anxiety and depression during COVID-19 [11].

In sum, college students' need for mental health care has increased dramatically [12,13]. Besides treatment, prevention is essential in order to substantially reduce the prevalence and incidence of mental disorders in the first place [13]. Haggerty and Mrazek [14] categorize prevention into three strategies. Universal prevention is directed at the general population without specific risk factors, and aims to bolster health promotion through resource enhancement for health maintenance. Selective prevention targets subgroups of the population at elevated risk of developing mental illnesses. Indicated prevention applies to individuals identified as high-risk for mental illness, who exhibit early indicators, symptoms, or biomarkers. This paper primarily focuses on the application of universal prevention strategies.

Prevention in college students can be very diverse and may address different areas such as depression, alcohol and addiction problems, but also problem-solving strategies or test anxiety [15]. Findings on the general effectiveness of mental health prevention among students are mixed. A meta-analysis demonstrated the effectiveness of skills-based programs with a supervisor [16]. Another umbrella meta-analysis reported that while universal interventions for depression, anxiety, and stress are available, their effectiveness varies widely. Effective interventions have been developed for smoking cessation, test anxiety, internet addiction, procrastination, and bystander sexual assault [15]. In a study of medical students participating in a seminar series on burnout and stress prevention, which was peer-led, 7.5 % were found to be at risk of burnout, 13 % were highly stressed, and 40 % showed symptoms of mild depression [17]. While the seminar series did not reduce these clinical symptoms, it was rated positively by the participants and considered to be relevant. To foster the effective utilization of mental health prevention services among students, it is crucial to understand the factors influencing their uptake. Although there is extensive research on intervention utilization, prevention-specific studies are less common. Drawing from the broader literature, it has been identified that factors such as stigma and negative beliefs about mental health services act as barriers, whereas positive past experiences and mental health literacy act as facilitators [18,19]. Nagai [20] suggested that depressive symptoms exert dual effects on help-seeking behavior. In the short term, they reduce motivation to seek help, but over time, persistent depressive symptoms distress individuals and drive them to seek professional assistance, increasing actual help-seeking behavior. Research on mental health problems in general has shown that personal attitudes and beliefs about mental health professionals and their ability to help also have a significant influence on help-seeking behavior [21].

The American College Health Association-National College Health Assessment (ACHA-NA, [22]), examining college students’ interest in learning about specific health topics, revealed that students were most interested in sleep difficulties (67.5 %), followed by physical activity (63.9 %) and suicide prevention (60.5 %). However, not all interested students received information on these topics. Findings regarding the preference for online or face-to-face counseling remain ambiguous. Online counseling offers more options and easier access, yet there appears to be a leaning towards face-to-face interaction [23,24]. In 2018, Toscos and colleagues [25] investigated students' willingness to use telemental health resources (TMH), including an anonymous chat with a trained nonprofessional, online therapy, and a set of self-help resources. The authors found that 24.6 %–40.1 % of the participants were willing to try TMH. Students generally preferred face-to-face over online resources, while students with elevated depression or anxiety scores were more likely and willing to use online chat sites and self-help resources [25]. Likewise, Moussa and Asender [24] found that the investigated college students had positive attitudes towards both face-to-face and online counseling, but showed a greater preference for face-to-face counseling. Particularly in view of the significant restrictions on social contact imposed during the COVID-19 pandemic, we hypothesized that students may favor direct interaction.

The main goal should be not only to achieve effectiveness of prevention programs, but also to increase students' uptake and utilization of such programs. To the best of our knowledge, there is a dearth of research examining students' interest and preferences with respect to mental health prevention. In the present study, we therefore aimed to address this gap by analyzing students' willingness to use mental health prevention, ascertaining what topics are of most interest to them, and determining whether men and women differ in their general interest in mental health prevention. In addition, we measured students' depression, anxiety, resilience, and coping levels during the pandemic and analyzed the relationship between students' mental health and their level of interest in and preferences regarding prevention. In their review, Magaard and colleagues [26] found that within seven of the sixteen datasets analyzed, there was a positive relation between the severity of depression and the frequency of help-seeking behavior. Consistent with these findings, Montagni et al. [27] indicated that based on the studies reviewed, e-mental health is currently being used for support seeking almost exclusively by students experiencing a mental health problem or disorder. Furthermore, Hämäläinen and colleagues [28] reported that the level of functional impairment in depression as well as anxiety disorder had an independent effect on the use of health services. Consequently, we hypothesized that participants with elevated anxiety and depression scores would show a greater interest in prevention than participants with scores in the normal range.

## 2 Methods

### 2.1 Sample

The present study was conducted between September 14 and October 18, 2021. It is a follow-up survey of a study conducted in 2020 by the Department of Psychology of the Julius-Maximilian-University of Würzburg [29]. For the initial study by Hein and colleagues [29], recruitment e-mails were sent to all enrolled students of the Julius-Maximilian-University of Würzburg, the University of Applied Sciences Würzburg-Schweinfurt, and the University of Music Würzburg. These are all state-funded universities, where students are not required to pay any fees. At the end of this survey, participants were asked whether they were willing to be contacted again for follow-up surveys. Accordingly, 3145 students were invited via email to participate in the present study. Participants were asked to complete an online questionnaire on a voluntary basis. All participants provided informed consent. As compensation, participants were entered into several raffles to win 50 Euros, with about 5 % of the participants winning this amount. The study was approved by the ethics committees of the Department of Psychology, University of Würzburg, and of the Medical Faculty, University of Freiburg and was conducted in accordance with the tenets of the Declaration of Helsinki.

### 2.2 Measures

#### 2.2.1 Demographic and medical characteristics

Participants reported their gender, age (in years), semester of study, country of citizenship, and their COVID-19 vaccination status as a medical characteristics.

#### 2.2.2 Depressive symptoms

Depression was assessed using the Patient Health Questionnaire-2 (PHQ-2; [30]. Students reported how often they had experienced these two depressive symptoms (depressed mood and anhedonia) in the last two weeks on a four-point Likert scale (0 = *not at all*, 3 = *nearly every day*). Total scores range from 0 to 6, with a score of ≥3 indicating a major depressive disorder [31].

#### 2.2.3 Anxiety symptoms

To assess anxiety, participants completed the 18-item Anxiety Sensitivity Index-3 (ASI-3; [32], which assesses the three domains (six items each) that are most indicative of anxiety sensitivity (AS): social, cognitive, and physical [33]. Items are rated on a five-point Likert scale (0 = *strongly disagree*, 4 = *strongly agree*); each scale score results from the sum of the raw scores of the six items and ranges from 0 to 24. The ASI-3 total score ranges from 0 to 72, with scores ≥25 considered indicative of anxiety sensitivity [34].

#### 2.2.4 Resilience

Resilience was assessed with the 10-item Connor-Davidson Resilience Scale (CD-RISC-10). The items measure the ability to bounce back from the variety of challenges that can arise in life [35]. Scores range from 0 to 40, and scores below a cut-off ≤23 are considered clinically relevant [36].

#### 2.2.5 Interest in mental health prevention

First, students were asked to rate their interest in mental health topics in general on a seven-point Likert scale (1 = *not interested at all*, 7 = *very interested*). Second, to establish which topics students were particularly interested in, they were asked to rate their interest in the following mental health topics on a seven-point Likert scale (1 = *not interested at all*, 7 = *very interested*): mental disorders, self-harm behaviors, emotion regulation, self-compassion, mindfulness, promotion of resilience, and training for interacting with stressed fellow students.

#### 2.2.6 Willingness to use mental health prevention resources

The survey contained six questions on students' willingness to use prevention resources. On a seven-point Likert scale (1 = *not at all*, 7 = *very likely*), students rated how likely they would be to use the following prevention resources: an online course encompassing 4–8 units on different topics, an online course encompassing one unit on a personally chosen topic, a face-to-face workshop encompassing 4–8 units on different topics, a face-to-face workshop encompassing one unit on a personally chosen topic, a weekly seminar on several topics, and information brochures.

#### 2.2.7 Knowledge of crisis contact options

In addition, students were asked whether they knew where to turn in the event of a mental health crisis experienced by themselves or a friend (*yes*/*no*).

#### 2.2.8 Coping measures taken during the pandemic

Participants were also asked what actions they took to strengthen their own mental health or maintain their quality of life during the pandemic. Specifically, they were asked whether they had engaged in the following activities (yes/no): sports, new hobby, digital contact with family or friends, practical tasks, craft projects, spending time with a pet.

### 2.3 Statistical analyses

Analyses were performed with R version 4.0.4 (2021-02-15) and R Studio (version 2022.02.3 + 492) using de-identified participant information. To investigate group differences, t-tests were used for continuous data and the Wilcoxon signed-rank test was used for ordinal data. In the case of significant differences, effect sizes were calculated using Cohen's *d* for t-tests and the Pearson correlation coefficient for the Wilcoxon signed-rank test. Effects were considered statistically significant at an alpha <0.05. In order to predict the students' general interest in mental health prevention from the aforementioned variables, we conducted a multiple regression.

## 3 Results

### 3.1 Demographic and medical characteristics

A total of 1334 participants were included, consisting of 70.8 % females and 29.2 % males, and the mean age was M = 24.8 years (SD = 3.33 years). The response rate was approximately 42 %. Participants reported being in their third to 22nd semester of study, with a mean of 6.55 semesters (*SD* = 2.71 semesters). All participants were university students and the majority were German citizens (97.8 %) and fully vaccinated (93.3 %; note: this equaled two doses at that time).

### 3.2 Measures of mental health

Means and standard deviations for the measures of mental health are presented in Table 3. The survey revealed that 28.11 % of the participants exhibited elevated levels of depression, 37.33 % had elevated anxiety sensitivity scores, and 38.98 % had clinically relevant resilience scores in terms of having low resilience/limited coping strategies. PHQ-2 scores did not significantly differ between males and females, *t*(1332) = 1.50, *p* = .133. However, a significant difference emerged regarding the ASI-3 total score, insofar as men showed lower scores compared to women, *t*(1332) = −1.98, *p* = .048, with a small effect size of *d* = 0.12. Furthermore, women had significantly lower CD-RISC-10 scores than did men, *t*(1332) = 4.21, *p* < .001, with a small effect size of *d* = 0.25.

Participants with elevated PHQ-2 scores showed a significantly higher general interest in mental health prevention offers compared to those with scores in the normal range, *t*(1318) = 5.56, *p* < .001, *d* = 0.34. The same was true for ASI-3 total scores *t*(1318) = −6.12, *p* < .001, *d* = 0.35. With regard to CD-RISC-10 scores, a significant difference in the reported general interest emerged between participants with clinically relevant scores and those with normal scores, pointing to more interest among students with lower resilience *t*(1318) = 4.41, *p* < .001, *d* = 0.25 (see Table 4).

### 3.3 Interest in mental health topics and the willingness to use mental health prevention

Participants' overall interest in information on mental health topics showed a mean of 5.58 (*SD* = 1.47) on the 7-point scale. As depicted in Table 1, students expressed most interest in emotion regulation (*M* = 5.30, *SD* = 1.68), followed by promotion of resilience (*M* = 5.29, *SD* = 1.75). Students were least interested in information on self-harm behaviors (*M* = 4.21, *SD* = 1.94). There was a statistically significant difference in the reported general interest between males and females, insofar as men were less interested in mental health prevention, *t*(1318) = −8.64, *p* < .001, with a medium effect size of *d* = 0.52.

**Table 1:** Interest in mental health topics for total sample and stratified for gender with group difference statistic.

	Total Sample	Male	Female
	*M* (*SD*)	*M* (*SD*)	*M* (*SD*)
General interest	5.58 (1.47)	5.05 (1.63)	5.80 (1.34)
Emotion regulation	5.30 (1.68)	4.70 (1.81)	5.55 (1.56)
Resilience	5.29 (1.75)	4.86 (1.81)	5.47 (1.69)
Mindfulness	4.94 (1.84)	4.44 (1.94)	5.14 (1.75)
Self-compassion	4.82 (1.75)	4.26 (1.84)	5.05 (1.66)
Mental disorders	4.82 (1.82)	4.36 (1.80)	5.01 (1.79)
Coping with stressed students	4.31 (1.96)	3.92 (1.90)	4.47 (1.97)
Self-harm	4.21 (1.94)	3.98 (1.89)	4.30 (1.96)

Note. Items range from 1 = “not interested at all” to 7 = “very interested”.

Of the prevention resources presented, respondents showed the greatest willingness to use an online program encompassing one unit on a topic of their own choice (*M* = 4.37, *SD* = 1.90). Students showed the lowest willingness to participate in a weekly seminar on several topics (*M* = 3.02, *SD* = 1.87). A paired sample *t*-test demonstrated that the willingness to use an online course comprising 4–8 units on different topics was significantly higher than the willingness to use a face-to-face workshop comprising 4–8 units on different topics (*t*(1313) = 8.704, *p* < .001, *d* = 0.24).

### 3.4 Knowledge of crisis contact options and coping measures taken during the pandemic

In total, 47.6 % of the participants reported that they knew whom to contact in the event of a mental health crisis concerning themselves or a friend, meaning that 52.4 % were unaware of such options. Participants had taken a mean of 2.70 (*SD* = 1.35) out of seven presented actions to strengthen their mental health during the pandemic. Men engaged in significantly fewer activities than did women, *t*(1332) = −6.98, *p* < .001 (see Table 2).

**Table 2:** Actions taken to strengthen mental health during the pandemic.

	N	%
contact with family or friends	990	74.2 %
sports	864	64.8 %
organizing or cleaning out	667	50.0 %
craft projects	369	27.7 %
new hobby	348	26.1 %
other	191	14.3 %
pet	168	12.6 %

**Table 3:** Means and standard deviations for mental health measures.

	M (SD)	Minimum	Maximum
**PHQ-2**	2.0 (1.7)	0	6
**ASI-3 total**	21.7 (12.5)	0	72
**CD-RISC-10**	24.8 (6.8)	0	40

**Table 4:** Linear regression table for general interest in prevention.

	**Coefficients**				
	**Non-standardized**		**Standardized**	**t-value**	**CI**^b^
	B	SE^a^	beta		
Constant	4.371	0.46		9.59∗∗∗	3.55–5.51
Number of activities	0.169	0.03	1.11	5.56∗∗∗	0.13–0.26
Depression score	0.117	0.03	1.59	4.23∗∗∗	0.06–0.18
Physical anxiety	0.006	0.01	1.49	0.67	−0.02-0.02
Social anxiety	−0.015	0.01	1.41	−1.69∗	−0.02-0.01
Cognitive anxiety	0.044	0.01	2.04	4.59∗∗∗	0.02–0.06
Resilience	−0.001	0.01	1.55	−0.15	−0.02-0.01
Contact options	−0.240	0.08	1.04	−3.02∗∗∗	−0.35-0.00
Age	−0.015	0.01	1.03	−1.24	−0.05-0.01
Gender (reference male)	0.621	0.09	1.10	6.72∗∗∗	0.40–0.81
Vaccination	0.037	0.07	1.02	0.57	−0.11-0.17

Adjusted R^2^ = 0.131; F Statistic = 20.493∗∗∗ (df = 10; 1287).

a *robust SE using HC3 due to homoscedasticity*.

b *Confidence Intervals [2.5%-97.5 %] Bootstrapping with R= 5000*; *∗p < 0.1;* *∗∗p < 0.05;* *∗∗∗p < 0.01*. *Physical anxiety sensitivity (fear that physical sensations are a sign of an immediate physical problem); Social anxiety sensitivity (fear of showing symptoms in public that could lead to embarrassment); Cognitive anxiety sensitivity (fear of losing cognitive control or having difficulty concentrating); Resilience is the ability to tolerate change, personal problems, illness, pressure, failure, and painful feelings; Contact options: Do I know where to get help.*

### 3.5 Multiple regression model

The overall model was statistically significant (*F*(10; 1287) = 20.493^,^ *p* < .001) and explained 13 % of the variance (adjusted R^2^ = 0.131). Significant predictors in the model were the number of activities students engaged in to maintain their mental health, their individual depression score, their social and cognitive anxiety sensitivity, the number of contact options they were aware of during the pandemic, and gender. As not all statistical requirements were fulfilled, we used robust estimates as well as confidence intervals generated with bootstrapping in R (samples = 5000). In terms of content, the effects can be interpreted as follows: The number of activities engaged in during the pandemic, the depression score, and physical and cognitive anxiety sensitivity increased the students' general interest in mental health prevention. On the other hand, social anxiety sensitivity and a higher number of contact options decreased it. The control variables showed that only gender had a statistically significant influence, with females showing a greater interest than males.

## 4 Discussion

The recent pandemic has highlighted the importance of personal resources and resilience in maintaining mental health. The present research, corroborated by global data, suggests that students face numerous challenges and often struggle with mental health issues, requiring appropriate responses from universities. Our findings indicate that female students suffered more mental health issues but also engaged more in mental health activities. Students who scored above a cut-off for clinical depression [31] and had higher dimensional anxiety scores showed a greater interest in mental health resources. The preference for online prevention methods points to a potential shift in how mental health services might be delivered effectively to this group. Moreover, the results emphasize the importance of customized support for students, considering gender differences and preferences for digital platforms.

### 4.1 Mental health

Consistent with previous findings, a large proportion of our sample reported mental health problems [8,11,37]. On each of the psychopathology scales, about a third of the participants had clinically relevant scores, with female students showing significantly more problems than males. The same pattern of findings has been reported in previous studies, indicating that females are more likely than males to suffer from anxiety and depressive disorders and to show lower resilience [[38], [39], [40]]. Nevertheless, in the context of our study, the PHQ-2 scores did not significantly differ between males and females, which diverges from the commonly held belief that there are notable gender differences in the prevalence of depressive symptoms [[41], [42], [43]]. This finding is supported by other studies [44,45]. Arcand and colleagues [44] suggested that the pandemic may have had a unique psychological impact on males, potentially exacerbating depressive symptoms to a level that parallels those typically reported by females. Moreover, not only young women, but women as a whole, have been disproportionately affected by the pandemic in terms of their mental health [8,46], meaning that female students are particularly at risk.

A further important finding of the present study is that students with clinically relevant depression, anxiety, and resilience scores showed significantly more interest in mental health than did those with scores in the normal range. This strongly suggests that students need psychological support. Affected students evidently have a high level of interest in mental health topics, indicating that mental health prevention should be integrated into academic curricula. A World Health Organization mental health survey revealed that only 16.4 % of students with mental disorders received treatment [2], indicating an extreme lack of supply for students in general [47]. Students are less likely to receive treatment when stigma is attached to seeking mental health services.

### 4.2 Reported interest in mental health topics and the willingness to use mental health prevention

According to our survey, students reported a high level of interest in mental health prevention overall, and females reported significantly more interest than males. This finding is in line with previous studies on mental health literacy [22,48]. A possible explanation for this gender difference may lie in the personality trait of agreeableness [49], with women having been shown to be more interested in the plight of others due to greater empathy. This difference may not only indicate a divergence in actual interest levels but might also reflect a disparity in the opportunities or inclinations to express such interest. Moreover, the mental health questionnaires also revealed significant gender differences, with women showing significantly more mental health problems, thus potentially explaining their greater reported interest in mental health prevention. However, it is important to keep in mind that study participation was voluntary and might be biased towards individuals with a greater interest in student mental health. Furthermore, more women than men completed the online questionnaire, which is a typical finding for voluntary surveys [50,51].

The analysis of students' preferred mental health topics revealed that they were most interested in emotion regulation, resilience, mindfulness, and self-compassion. Thus, students showed a greater preference for strategies to strengthen their own resources over gaining knowledge about mental disorders or self-harm behaviors. This finding is quite interesting, as many prevention programs have psychoeducational elements and often focus on mental health problems instead of promoting mental stability and resilience [52,53]. In a meta-analysis examining the effectiveness of both published and unpublished universal prevention programs for college students, Conley and his colleagues [16] found that skill-training programs with supervised practice were the most effective [16], which is in line with the preferences expressed by the present sample: skills to improve emotion regulation, resilience, and self-compassion. Interestingly, resilience was identified as the most important predictor of the impact of the COVID-19 pandemic on students' mental health [39]. Resilience plays a crucial role, especially in higher education, as it is seen as a necessary key competence for professionals and managers in later professional life [54,55]. Unfortunately, there is little research on this specific area and on the question of how higher education institutions can help to foster students’ mental health and promote resilience in order to avoid negative consequences such as dropping out of their studies or developing burnout in the long term [56].

Furthermore, our sample preferred online prevention over face-to-face prevention. This finding is contrary to our hypothesis and to previous (pre-pandemic) studies that indicated a preference for face-to-face interventions or therapy [25]. However, the online nature of our questionnaire may have introduced a response bias in this regard, as it is possible that individuals who did not respond are more likely to have a stronger preference for face-to-face communication. Additionally, this discrepancy might be explained by the different focus of the surveys: Some focus on general issues that should possibly be addressed collectively, while others focus on individual issues that should possibly be clarified privately. Moreover, the timing of our survey may have exerted an influence on students’ responses and therefore the current results. As students were largely familiar with the use of online courses and services by autumn 2021, we can assume that any fears or aversions regarding online tools during the pandemic had reduced [57], which may in turn have increased the willingness to use online services.

### 4.3 Knowledge of crisis contact options and coping measures taken during the pandemic

The survey highlighted a significant information deficit, with over half of the respondents (52.4 %) unaware of whom to contact during a mental health crisis. This finding highlights the critical need for improved educational and communication efforts to ensure that individuals are aware of the support available to them. Additionally, the data suggest a subdued participation in proactive mental health measures during the pandemic. It is particularly noteworthy that, despite being more impacted by mental health issues, female students took part in a greater number of activities aimed at maintaining their mental health compared to their male counterparts.

### 4.4 Implications

The findings of this study are highly relevant to the current global context, especially considering the impact of the COVID-19 pandemic on student mental health and the shift to online learning.

The disruption of education due to the pandemic, with a swift shift to online learning and associated stress, has deeply affected student mental health, emphasizing the critical need for robust mental health support and preventive measures. The study's findings that students, particularly females, proactively engage in activities to enhance their mental health and demonstrate a distinct preference for online mental health prevention methods are particularly noteworthy. This implies that the transition to online learning may have also shaped students' preferences for receiving mental health support. Reviewed studies [27] underline the high demand among students for online mental health information, which remains consistent regardless of their mental health status.

Existing programs like the Be Well Plan, a 5-week online intervention for students, have been effective in enhancing mental health and resilience and reducing depression and anxiety, with high satisfaction and engagement levels reported [58]. Another prevention program is "Talk to me", a mass open online course (MOOC) aiming to improve mental health literacy among students and those close to them [59]. The program was well-received, significantly boosting participants' self-efficacy and knowledge on managing suicidal thoughts [60]. These findings underscore the potential of internet-based, group-facilitated interventions in addressing mental health issues among university students. Considering the barriers of mental health-related digital use, Montagni et al. [27] emphasized the importance of creating youth-friendly digital mental health tools with clear data privacy, high-quality content, and easy navigation. Mandatory MOOCs could be an effective initial approach, offering a safe, bullying-free home learning environment, covering various topics with clear assistance. Subsequent personalized courses like workshops could further bolster students’ mental strength and lifelong resilience. Furthermore, this study highlights the critical need for mental health resources that are specifically tailored to meet the individual needs and preferences of students, taking into account factors such as gender differences. This underscores the message for universities and mental health practitioners that a one-size-fits-all approach may not suffice. Instead, there is a need for personalized mental health resources that meet the unique needs of diverse student groups.

In sum, this study provides valuable insights that can guide universities and mental health professionals in developing and implementing effective mental health support strategies in the current global context. It highlights the importance of resilience, the need for tailored resources, and the potential of online platforms in addressing student mental health issues in the wake of the COVID-19 pandemic.

### 4.5 Limitations

This study has some limitations that should be considered and discussed. First, while our results are based on a relatively large sample of German college students, the sample cannot be considered as representative for German college students or college students worldwide.

Second, as mentioned above, gender is unequally distributed in our sample, meaning that our results cannot be generalized due to the overrepresentation of women. Since men and women may have different responses or behaviors, the preponderance of women might skew the findings towards female patterns of behavior or responses. Recent studies, for example, found gender differences regarding reactions to the COVID-19 pandemic [[61], [62], [63]].

Moreover, it would have been interesting to include the participants' field of study in our survey, as there is evidence that students' mental health differs depending on their field of study or faculty [64]. It may likewise have been illuminating to consider the participants' semester of study in the statistical analyses, since the semester that a student is in can also significantly influence their experiences and perspectives. However, due to the significant number of missing values, we chose to discard this option. Freshmen, who are typically adjusting to a new environment and academic expectations, might face different stressors compared to seniors, who may be dealing with the pressures of impending graduation and future career plans. Therefore, not considering the participants’ field of study or semester limits the generalizability of the study results, as the findings might not accurately reflect the experiences and perspectives of all college students. Future studies could benefit from considering this factor in order to provide a more comprehensive understanding of the phenomena under investigation.

### 4.6 Future research

In view of the findings of our study, several avenues for future research can be suggested. First, it would be beneficial to explore the factors that contribute to the high interest in mental health prevention among students, particularly those with clinically relevant scores. Understanding these factors could help inform the development of preventive measures that effectively address students' needs and preferences. Second, the gender differences observed in our study warrant further investigation. Future research could explore why females engage in more activities to strengthen their mental health and why they exhibit higher anxiety scores and lower resilience scores compared to their male counterparts. Such research might provide insights into gender-specific risk factors and protective factors, which could inform the development of gender-sensitive preventive measures. Third, given the high motivation but low knowledge about where to find professional support, future research could investigate the barriers that prevent students from accessing mental health services. In the light of the relatively sparse body of research on the barriers and facilitators for preventive measures, it is crucial to investigate these aspects. Such exploration could significantly enhance our understanding and implementation of effective prevention strategies in mental health. Lastly, our findings highlight the need for easily accessible and low-threshold mental health prevention offers to be integrated into university curricula. Future research could evaluate the effectiveness of such initiatives in improving students’ mental health and resilience.

This area of research offers numerous avenues for exploration and has the potential to significantly enhance our understanding of prevention in the context of today's rapidly evolving academic landscape.

## 5 Conclusion

Our results indicate that a substantial proportion of college students appear to suffer from mental health problems. This alarming finding underlines the clear need for effective prevention strategies to avoid long-term social, educational, and occupational impairment [65]. Experts argue that college students should be prime targets for mental health prevention, as many mental health problems occur during the college years [16] and have significant consequences throughout an individual's life [66,67]. Our findings underline the responsibilities of higher education institutions in this regard and suggest a clear need for action. As such, mandatory seminars or courses may be required in the initial semesters to build knowledge, reduce stigma, and enhance functional coping strategies. Universities should establish effective action plans to promote students' resilience, and gender-specific prevention also needs to be integrated into academic curricula [68]. To enable a larger number of students to benefit from prevention services, barriers to help-seeking need to be reduced. The present survey, examining students' preferences and willingness with respect to mental health prevention, represents a first step in this direction, but further steps lie ahead.

## CRediT authorship contribution statement

**Lena Ehl:** Writing – review & editing. **Christin Scheiner:** Writing – original draft, Formal analysis, Conceptualization. **Antonia Wasserscheid:** Writing – review & editing. **Grit Hein:** Writing – review & editing, Project administration, Methodology, Data curation. **Matthias Gamer:** Writing – review & editing, Project administration, Methodology, Data curation. **Arne Bürger:** Writing – review & editing, Writing – original draft, Supervision, Conceptualization.

## Ethics

This study is a secondary data analysis with no human subject issues. The ethics statement is included in the paper.

## Data availability

We do not have permission to share data.

## Transparency declaration

The manuscript is an honest, accurate, and transparent account of the study being reported. No important aspects of the study have been omitted. Any discrepancies from the study as planned (and, if relevant, registered) have been explained.

## Funding

This work was funded by the 10.13039/501100001663VolkswagenStiftung (AZ 99451) and the German Research Foundation (DFG 44541416-TRR58). The publication was supported by the Open Access Publication Fund of the 10.13039/501100008769University of Würzburg.

## Declaration of competing interest

The authors declare that they have no known competing financial interests or personal relationships that could have appeared to influence the work reported in this paper.
