# Supplementary material for: German college students’ mental health state and their willingness to use mental health prevention: An online survey during the COVID-19 pandemic
Source: Heliyon. 2025 Jan 31;11(3):e42290. doi: 10.1016/j.heliyon.2025.e42290 (PMC11849601; doi:10.1016/j.heliyon.2025.e42290)
Supplement: Multimedia component 1 [file mmc1.docx]

Welcome to the online questionnaire on emotional stress related to the Coronavirus (COVID-19) pandemic.

This study is conducted by the Department of Psychology I and the Center for Mental Health at the University of Würzburg.

**Objective of the Study:**

You have been contacted because you participated in the first part of this study in spring 2020. With this survey, we aim to investigate how various psychological parameters have developed since then. The goal of this research is to understand how individuals emotionally respond to the Coronavirus (COVID-19) pandemic. To achieve this, we kindly ask you to answer questions related to current concerns and anxieties, social support, as well as your current professional and personal life situation.

**Estimated Duration:**

Completing the entire questionnaire will take approximately 30 minutes.

**Voluntary Participation:**

Participation is voluntary. You can end your participation at any time without any disadvantages. In that case, simply close the browser window, and your data will be marked as incomplete and deleted at the end of the survey.

**Compensation**:

As a token of appreciation for participating, we will hold a prize draw among all participants who complete the questionnaire. Multiple winners will receive €50 each, so approximately every 20th participant will win. Participation will not be otherwise compensated, except through the chance to win in the prize draw.

If you wish to participate in the prize draw, please provide us with your email address so that we can inform you in case you win. Your email address will not be shared with third parties and will be stored separately from your survey data to ensure anonymity. The email address will be stored solely for this purpose and will be completely deleted by March 31, 2022. For the purpose of prize payout, personal data (including IBAN, full address, first name, and last name) will be collected and forwarded to the University of Würzburg’s Financial Service Center. These data will not be merged with the scientific dataset (survey data) and will only be used for the purpose of compensating participants in the study. If annual earnings from study participation exceed €1500, the university is obligated to report these earnings to the tax authorities; otherwise, no further action will be taken."

**Pseudonymization**:

The data collection is done pseudonymously. By using pseudonyms, we can link the data from the previous survey to the current one. This allows us to track changes in your responses without knowing your identity. All personal data, such as your name and address, are strictly separated from the survey data and are not disclosed to the researchers conducting the scientific analyses. Direct identification of individuals is thus excluded. With the help of your pseudonym, it is possible to delete the information you provided even after the fact. Please keep your pseudonym safe for this purpose. If you wish to revoke your participation or request partial or complete deletion of your data, please contact the study management.

**Data Privacy:**

The pseudonymized data collected will be scientifically evaluated, summarized into groups, and securely stored on servers for a minimum of 10 years in accordance with the EU General Data Protection Regulation. Additionally, they may be made publicly accessible in fully anonymized form via the Open Science Framework internet database to ensure good scientific practice. For more information on the processing of personal data and GDPR, you can refer to the University of Würzburg’s Data Protection Officer’s page.

**Data Deletion:**

During the survey, a personal code word will be generated. This code word does not allow any conclusions about your identity. However, using this code word, you can request the deletion of your data during the retention period by contacting the study management.

Furthermore, your code word allows us to combine your data from this survey with your data from the initial survey, enabling us to track changes in your responses without knowing your identity.

**Consent**:

Are you in agreement with these conditions and would like to participate in the study?

If you choose not to participate, please close the browser window now.

Yes, I agree and would like to participate."

**Instructions for creating a pseudonym:**

With the information, you provide on this page, a code word (pseudonym) will be generated. After completing the study, you can request the deletion of your data by providing the code word (e.g., ‘raul13091990’). Additionally, using your code word, we can combine your data from this survey with your data from the initial survey.

Please make sure to follow the instructions carefully for creating the code word.

What are the last two letters of your mother’s first name?

*Example: For “Petra Anna Schmidt,” the answer would be “ra.” If unknown or if you prefer not to provide this information, use “xx.”*

What are the last two letters of your father’s first name?

___________________________________________________________________________

*Example: For “Paul Peter Schmidt,” the answer would be “ul.” If unknown or if you prefer not to provide this information, use “xx.”*

What is your birthdate

___________________________________________________________________________
