# Supplementary material for: German college students’ mental health state and their willingness to use mental health prevention: An online survey during the COVID-19 pandemic
Source: Heliyon. 2025 Jan 31;11(3):e42290. doi: 10.1016/j.heliyon.2025.e42290 (PMC11849601; doi:10.1016/j.heliyon.2025.e42290)
Supplement: Multimedia component 2 [file mmc2.pdf]

Willkommen zum Online-Fragebogen über emotionale Belastungen infolge der Coronavirus (COVID-19) Pandemie

Diese Studie wird vom Lehrstuhl für Psychologie I und dem Zentrum für Psychische Gesundheit der Universität Würzburg in Kooperation mit der Clinical and Health Psychology-Arbeitsgruppe der Erasmus-Universität Rotterdam durchgeführt.

Ziel der Untersuchung

Ziel dieser Untersuchung ist es, ein Verständnis dafür zu entwickeln, wie Individuen emotional auf die Coronavirus (COVID-19) Pandemie reagieren. Zu diesem Zweck möchten wir Sie darum bitten, einige Fragen zu aktuellen Sorgen und Ängsten, sozialer Unterstützung, Mediennutzung sowie Ihrer momentanen beruflichen und persönlichen Lebenssituation zu beantworten.

Bearbeitungsdauer

Die Bearbeitung des kompletten Fragebogens wird **circa 20 Minuten** in Anspruch nehmen.

Freiwilligkeit und Datenschutz

Die Teilnahme ist freiwillig. Sie können die Teilnahme jederzeit beenden, ohne dass Ihnen daraus Nachteile entstehen. Schließen Sie in diesem Fall einfach das Browserfenster, es werden dann keine Daten gespeichert. Die Erhebung der Daten erfolgt vollständig anonymisiert, wir können also nicht rückschließen, wer Sie sind. Die Daten werden wissenschaftlich ausgewertet, in Gruppen zusammengefasst veröffentlicht und für einen Zeitraum von mindestens 10 Jahren in Übereinstimmung mit der Datenschutz-Grundverordnung der EU auf sicheren Servern aufbewahrt. Zudem können sie in vollständig anonymisierter Form über die Internet-Datenbank Open Science Framework zur Sicherstellung guter wissenschaftlicher Arbeit öffentlich zugänglich gemacht werden. Bitte beachten Sie hierzu auch die Informationen zur Verarbeitung personenbezogener Daten sowie zur DSGVO, die Sie über die Seite des Datenschutzbeauftragten der Universität Würzburg einsehen können.

Löschung der Daten

Im Rahmen der Befragung wird ein persönliches Codewort erstellt. Das Codewort erlaubt keine Rückschlüsse darauf, wer Sie sind. Mit diesem Codewort können Sie allerdings jederzeit die Löschung Ihrer Daten während des Aufbewahrungszeitraums verlangen. Außerdem können wir bei einer geplanten freiwilligen Nachbefragung nach ca. 6 Monaten mit Hilfe des Codeworts die erste und die zweite Erhebung zusammenfügen, um so Veränderungen in Ihren Antworten nachvollziehen zu können, ohne Ihre Identität zu kennen.

Einverständniserklärung

Sind Sie mit diesen Bedingungen einverstanden und möchten an der Studie teilnehmen?

Falls Sie nicht teilnehmen möchten, schließen Sie jetzt bitte das Browser-Fenster.

- ○ Ja, ich bin einverstanden und möchte teilnehmen.

Seite 02

1. Erstellung eines Pseudonyms

Mit Ihren Angaben auf dieser Seite wird ein Codewort (Pseudonym) erstellt. Sie können nach Beendigung der Studie die Löschung Ihrer Daten unter Angeben des Codeworts (z.B. raul13091990) verlangen.

Wie lauten die beiden letzten Buchstaben des (ersten) Vornamens Ihrer Mutter?	Beispiel: **ra** bei Pet**ra** Anna Schmidt. **xx** falls unbekannt oder falls Sie keine Angabe machen möchten.
Wie lauten die beiden letzten Buchstaben des (ersten) Vornamens Ihres Vaters?	Beispiel: **ul** bei Pa**ul** Peter Schmidt. **xx** falls unbekannt oder falls Sie keine Angabe machen möchten.
Was ist Ihr Geburtsdatum?	

Seite 03

2. Was ist Ihr Geschlecht?

- männlich
- weiblich
- divers
- keine Angabe

3. Was ist Ihre Staatsbürgerschaft?

Bei Mehrstaatigkeit bitte die Staatsbürgerschaft angeben, in dessen Staat Sie im vergangenen Jahr die meiste Zeit verbracht haben.

4. In welchem Land und Bundesland/Kanton befinden Sie sich im Moment hauptsächlich?

Land:	
Bundesland/Kanton:	

Seite 04

5. Wie viele Menschen leben aktuell in Ihrem Haushalt (Sie eingerechnet)?

Anzahl: ☐

6. Wer lebt mit Ihnen aktuell im Haushalt?

(Mehrfachauswahl möglich)

- ☐ (Ehe-)Partner*in
- ☐ Kinder, 0 bis 6-jährig
- ☐ Kinder, 7 bis 17-jährig
- ☐ Kinder, 18-jährig und älter
- ☐ Eltern und/oder Großeltern
- ☐ WG-Mitbewohner/Geschwister
- ☐ Haustiere
- ☐ Sonstige

- ☐ Nur ich

7. Bis zu welchem Ausmaß waren oder sind Menschen in Ihrem Umfeld am Coronavirus erkrankt?

	Leichter Verlauf	Schwerer Verlauf	Todesfall	Keine bekannten Fälle
Im privaten Umfeld (Familie oder enge Freunde/Bekannte)	○	○	○	○
Im beruflichen/studentischen Umfeld	○	○	○	○

8. Welche Tätigkeit üben Sie aktuell aus?

(Mehrfachauswahl möglich)

- ☐ Studium
- ☐ Ausbildung (bitte angeben): ______
- ☐ Erwerbstätigkeit, auch Teilzeit oder Nebenjob (bitte angeben): ______
- ☐ Arbeitslos/Arbeitssuchend
- ☐ Sonstiges (bitte angeben): ______

Seite 05

9. Bitte machen Sie folgende Angaben zu Ihrem Studium:

An welchem Ort studieren Sie?

An welcher Hochschule/Universtität studieren Sie?

Was studieren Sie?
(Bei Doppelstudium alle Studiengänge angeben)

Welchen Abschluss hat Ihr aktueller Studiengang?
(z.B. Bachelor, Master, Staatsexamen)

In welchem Fachsemester sind Sie?

10. Die folgenden Fragen beziehen sich auf Ihr Studium oder Ihre Hochschule/Universität:

	Nein	Ja
Befinden Sie sich aktuell am Studienort?	○	○
Die Informationen meiner Hochschule/Universität auf deren Homepage zur Coronavirus (COVID-19) Pandemie kenne ich.	○	○
Ich werde von meiner Hochschule/Universität angemessen über die Auswirkungen der Coronavirus (COVID-19) Pandemie auf mein Studium informiert.	○	○
Meine Hochschule/Universität reagiert angemessen auf die Coronavirus (COVID-19) Pandemie.	○	○

11. Wie finanzieren Sie Ihr Studium hauptsächlich?

- ○ Eltern oder Angehörige
- ○ BAföG oder Stipendium
- ○ Selbst

Seite 06

12. Wie wird bzw. hat die Coronavirus (COVID-19) Pandemie Sie in den folgenden Bereichen beeinflusst?
Antworten Sie nach Ihrem Gefühl, ohne lange nachzudenken.

	Sehr negativ	Negativ	Neutral	Positiv	Sehr positiv
Körperliche Leistungsfähigkeit	○	○	○	○	○
Geistige/intellektuelle Leistungsfähigkeit	○	○	○	○	○
Psychische/emotionale Befindlichkeit	○	○	○	○	○
Soziale Kontakte	○	○	○	○	○
Finanzielle Situation	○	○	○	○	○
Mobilität	○	○	○	○	○
Zukunftspläne	○	○	○	○	○

13. Wie wird bzw. hat die Coronavirus (COVID-19) Pandemie Ihr Studium beeinflusst?
Antworten Sie nach Ihrem Gefühl, ohne lange nachzudenken.

	Sehr negativ	Negativ	Neutral	Positiv	Sehr positiv
Vorlesungen	○	○	○	○	○
Seminare	○	○	○	○	○
Übungen / Praktische Veranstaltungen	○	○	○	○	○
Abschlussarbeiten	○	○	○	○	○
Prüfungsleistungen	○	○	○	○	○
Studiendauer	○	○	○	○	○
Berufsaussichten	○	○	○	○	○
Kontakt mit Dozenten	○	○	○	○	○
Kontakt mit Studierenden	○	○	○	○	○
Externe Praktika	○	○	○	○	○

Seite 07

14. Wie ist Ihre Einstellung zur digitalen Lehre bzw. online Lehre?

	Stimme überhaupt nicht zu				Stimme völlig zu
Finde ich gut	○	○	○	○	○
Bringt mir mehr zeitliche Flexibilität	○	○	○	○	○
Verbessert die Qualität der Lehre	○	○	○	○	○
Verursacht mir zusätzliche Kosten	○	○	○	○	○
Mein Studium wird unpersönlicher	○	○	○	○	○
Erleichtert mir die Studienplanung	○	○	○	○	○
Ich habe die notwendige Ausstattung	○	○	○	○	○
Führt zu mehr Einzelkämpfertum	○	○	○	○	○
Erweitert meine digitale Kompetenz	○	○	○	○	○

15. Inwieweit treffen die folgenden Aussagen auf Ihren Tagesablauf der letzten Woche zu?

	Trifft überhaupt nicht zu				Trifft völlig zu
Ich hatte eine klare Tagesstruktur.	○	○	○	○	○
Ich hatte angenehme Aktivitäten.	○	○	○	○	○
Ich hatte unangenehme Aktivitäten.	○	○	○	○	○
Ich war körperlich aktiv.	○	○	○	○	○
Ich hatte berufliche oder studentische Aufgaben.	○	○	○	○	○
Ich betreute Kinder (nicht von Berufs wegen).	○	○	○	○	○
Ich hatte sonstige Pflichten.	○	○	○	○	○
Ich vermied alle unnötigen Aktivitäten.	○	○	○	○	○

16. Wie sehr treffen folgende Aussagen auf Ihre Arbeitsstelle zu?

	Trifft überhaupt nicht zu				Trifft völlig zu
Meine Arbeitsstelle ist aufgrund der Coronavirus (COVID-19) Pandemie gefährdet.	○	○	○	○	○
Meine Arbeitsstelle/Lohn wurde aufgrund der Coronavirus (COVID-19) Pandemie gekürzt.	○	○	○	○	○

Seite 08

17. Welche Online Media-Plattform nutzen Sie mindestens einmal pro Woche?

- ☐ Youtube
- ☐ Facebook
- ☐ Instagram
- ☐ Twitter
- ☐ Reddit
- ☐ Pinterest
- ☐ Instant Messaging Apps (z.B. WhatsApp, Snapchat, WeChat)
- ☐ Online Angebote von Zeitungen und Zeitschriften (z.B. zeit.de, spiegel.de, nytimes.com)
- ☐ Sonstige (Bitte angeben): __________

- ☐ Keine

18. Wie viel Zeit verbringen Sie durchschnittlich am Tag auf den angegeben Social Media-Plattformen insgesamt?

- ○ 0 – 60 Min.
- ○ 1 – 2 Std.
- ○ 2 – 4 Std.
- ○ > 4 Std.

19. Welche der folgenden Quellen nutzen Sie für die aktuellsten Nachrichten?

	Überhaupt nicht	Wenig	Mäßig	Häufig	Sehr häufig
Fernseher	○	○	○	○	○
Radio	○	○	○	○	○
Gedruckte Zeitungen/Zeitschriften	○	○	○	○	○
Nachrichten-Websites (z.B. spiegel.de, tagesschau.de, cnn.com)	○	○	○	○	○
Soziale Netzwerk-Websites (z.B. Facebook, Twitter, Reddit)	○	○	○	○	○
Video-Sharing-Websites (z.B. YouTube)	○	○	○	○	○
Video-Streaming-Plattformen (z.B. Netflix)	○	○	○	○	○
Freunde und/oder Familie (persönliche Interaktion)	○	○	○	○	○

20. Wie oft schauen Sie online nach Updates zur Coronavirus (COVID-19) Pandemie?

- ○ Nie
- ○ Alle paar Tage
- ○ Ein Mal pro Tag
- ○ Mehrmals pro Tag
- ○ Ein Mal pro Stunde
- ○ Mehrmals pro Stunde

Seite 10

21. Beantworten Sie die folgenden Fragen nach Ihrem Gefühl, ohne lange nachzudenken.

	Sehr wenig				Sehr stark
Wie wahrscheinlich ist es, dass Sie mit dem Coronavirus infiziert werden?	○	○	○	○	○
Wie wahrscheinlich ist es, dass jemand, den Sie kennen, mit dem Coronavirus infiziert wird?	○	○	○	○	○
Wie stark sind Sie mit Informationen über das Coronavirus konfrontiert?	○	○	○	○	○
Inwieweit sind Sie besorgt, bei einer Infektion mit dem Coronavirus schwer zu erkranken?	○	○	○	○	○
Inwieweit sind Sie besorgt, dass jemand, den Sie kennen, durch das Coronavirus schwer erkranken könnte?	○	○	○	○	○
Inwieweit hat die Bedrohung durch das Coronavirus Ihre Entscheidung beeinflusst, unter Leuten zu sein?	○	○	○	○	○
Inwieweit hat die Bedrohung durch das Coronavirus Ihre Reisepläne beeinflusst?	○	○	○	○	○
Inwieweit hat die Bedrohung durch das Coronavirus Ihre Anwendung von Sicherheitsverhalten (z.B. Handdesinfektion) beeinflusst?	○	○	○	○	○
Machen Sie sich Sorgen, aufgrund der Coronavirus (COVID-19) Pandemie Ihren Arbeitsplatz zu verlieren bzw. Ihr Studium nicht erfolgreich abschließen zu können?	○	○	○	○	○
Machen Sie sich Sorgen, aufgrund der Coronavirus (COVID-19) Pandemie in finanzielle Nöte zu geraten?	○	○	○	○	○
Fühlen Sie sich den Herausforderungen durch die Coronavirus (COVID-19) Pandemie gewachsen?	○	○	○	○	○
Sehen Sie Forschung seit der Coronavirus (COVID-19) Pandemie als mehr relevant an?	○	○	○	○	○
Sehen Sie Gesundheitsberufe seit der Coronavirus (COVID-19) Pandemie als mehr relevant an?	○	○	○	○	○

Seite 11

22. Wie sehr treffen folgende Aussagen auf Ihr Verhalten in den letzten 2 Wochen zu?

	Trifft überhaupt nicht zu				Trifft völlig zu
Wenn ich in den Lebensmittelladen ging, kaufte ich mehr Essen ein als sonst.	○	○	○	○	○
Ich zog es vor, zu Hause zu bleiben, um soziale Kontakte zu vermeiden.	○	○	○	○	○
Ich vermied Körperkontakt (z.B. Händeschütteln, Umarmungen).	○	○	○	○	○
Ich versuchte, das Berühren von potentiell kontaminierten Objekten zu vermeiden (z.B. Türklinken, Fahrstuhlknöpfe etc.).	○	○	○	○	○
Ich vermied soziale Aktivitäten wie Besuche von Restaurants, Theater, Kinos, etc.	○	○	○	○	○
Ich versuchte, einen Sicherheitsabstand zu Anderen einzuhalten.	○	○	○	○	○

23. Wie sehr treffen folgende Aussagen aktuell auf Sie zu?

	Trifft überhaupt nicht zu				Trifft völlig zu
Ich fühle mich durch mein soziales Umfeld (Familie, Freunde) unterstützt.	○	○	○	○	○
Ich habe Angst, aufgrund der Corona-Krise zu vereinsamen.	○	○	○	○	○
Positive Interaktionen mit anwesenden Personen entspannt mich.	○	○	○	○	○
Digitale Interaktionen können persönliche Interaktionen ersetzen.	○	○	○	○	○
Mein Mitgefühl gilt ausschließlich den Corona-Opfern in meinem Heimatland.	○	○	○	○	○
Die Corona-Krise hat meine Einstellung gegenüber anderen Nationen negativ beeinflusst.	○	○	○	○	○

Seite 12

24. Wie sehr stimmen Sie folgenden Aussagen zu?

	Stimme überhaupt nicht zu	Stimme nicht zu	Weder noch	Stimme zu	Stimme völlig zu
Ich habe Angst vor dem Coronavirus-19.	○	○	○	○	○
Es ist mir unangenehm, über das Coronavirus-19 nachzudenken.	○	○	○	○	○
Meine Hände werden klamm, wenn ich über das Coronavirus-19 nachdenke.	○	○	○	○	○
Ich habe Angst davor, wegen des Coronavirus-19 mein Leben zu verlieren.	○	○	○	○	○
Ich werde ängstlich und nervös, wenn ich Nachrichten über das Coronavirus-19 auf sozialen Medien sehe.	○	○	○	○	○
Ich kann nicht schlafen, weil ich mir Sorgen darüber mache, das Coronavirus-19 zu bekommen.	○	○	○	○	○
Mein Herz rast oder pocht, wenn ich darüber nachdenke, das Coronavirus-19 zu bekommen.	○	○	○	○	○

Seite 13

25. Wie fühlen Sie sich jetzt, in diesem Moment?

Geben Sie an, wie Sie sich **jetzt, in diesem Moment** fühlen. Es gibt keine richtigen und falschen Antworten. Überlegen Sie nicht lange und wählen Sie die Antwort, die Ihren augenblicklichen Gefühlszustand am besten beschreibt.

	Überhaupt nicht	Ein wenig	Ziemlich	Sehr
Ich bin ruhig.	○	○	○	○
Ich fühle mich angespannt.	○	○	○	○
Ich bin aufgeregt.	○	○	○	○
Ich bin entspannt.	○	○	○	○
Ich bin zufrieden.	○	○	○	○
Ich bin besorgt.	○	○	○	○

26. Wie sehr treffen folgende Aussagen auf Sie zu?

	Überhaupt nicht	Wenig	Teils teils	Sehr	Völlig
Unvorhergesehene Ereignisse regen mich sehr auf.	○	○	○	○	○
Es frustriert mich, wenn ich nicht alle Informationen habe, die ich brauche.	○	○	○	○	○
Unsicherheit hält mich davon ab, ein erfülltes Leben zu führen.	○	○	○	○	○
Man sollte immer nach vorne schauen, um Überraschungen zu vermeiden.	○	○	○	○	○
Ein kleines unvorhergesehenes Ereignis kann alles verderben, selbst bei bester Planung.	○	○	○	○	○
Wenn es Zeit zum Handeln ist, lähmt mich die Unsicherheit.	○	○	○	○	○
Wenn ich unsicher bin, kann ich nicht sehr gut funktionieren.	○	○	○	○	○
Ich möchte immer wissen, was die Zukunft für mich bereithält.	○	○	○	○	○
Ich kann es nicht ertragen, wenn man mich überrascht.	○	○	○	○	○
Der kleinste Zweifel kann mich vom Handeln abhalten.	○	○	○	○	○
Ich sollte in der Lage sein, alles im Voraus zu organisieren.	○	○	○	○	○
Ich muss aus allen unsicheren Situationen herauskommen.	○	○	○	○	○

Seite 15

27. Wie typisch oder charakteristisch sind folgende Aussagen für Sie?

	Überhaupt nicht typisch	Nur wenig typisch	Ziemlich typisch	Sehr typisch	Äußerst typisch
Wenn ich nicht genug Zeit habe, alles zu erledigen, mache ich mir darüber keine Sorgen.	○	○	○	○	○
Meine Sorgen wachsen mir über den Kopf.	○	○	○	○	○
Ich neige nicht dazu, mir über Dinge Sorgen zu machen.	○	○	○	○	○
Viele Situationen machen mir Sorgen.	○	○	○	○	○
Ich weiß, ich sollte mir keine Sorgen machen, aber ich kann nichts dagegen machen.	○	○	○	○	○
Wenn ich unter Druck stehe, mache ich mir viel Sorgen.	○	○	○	○	○
Über irgend etwas mache ich mir immer Sorgen.	○	○	○	○	○
Mir fällt es leicht, sorgenvolle Gedanken zu vertreiben.	○	○	○	○	○
Sobald ich eine Aufgabe beendet habe, fange ich an, mir über all das Sorgen zu machen, was ich sonst noch tun muß.	○	○	○	○	○
Ich mache mir nie über etwas Sorgen.	○	○	○	○	○
Wenn ich in einer Angelegenheit nichts mehr tun kann, mache ich mir auch keine Sorgen mehr darüber.	○	○	○	○	○
Ich war schon immer jemand, der sich viel Sorgen macht.	○	○	○	○	○
Mir fällt auf, daß ich mir über einiges Sorgen gemacht habe.	○	○	○	○	○
Wenn ich erst einmal anfange, mir Sorgen zu machen, kann ich nicht mehr damit aufhören.	○	○	○	○	○
Ich mache mir die ganze Zeit über Sorgen.	○	○	○	○	○
Ich mache mir über Vorhaben solange Sorgen, bis sie komplett erledigt sind.	○	○	○	○	○

28. Zu welchem Grad stimmen Sie folgenden Aussagen zu?

Sollte eine Aussage Inhalte thematisieren, die Sie nicht erlebt haben, antworten Sie bitte gemäß der Erwartung, wie Sie sich bei einer solchen Erfahrung fühlen würden.

	Sehr wenig	Wenig	Teils teils	Ziemlich	Sehr stark
Es ist mir wichtig, nicht nervös zu erscheinen.	○	○	○	○	○
Wenn ich mich nicht auf eine Aufgabe konzentrieren kann, befürchte ich, verrückt zu werden.	○	○	○	○	○
Es macht mir Angst, wenn ich starkes Herzklopfen verspüre.	○	○	○	○	○
Wenn ich mir den Magen verdorben habe, befürchte ich, dass ich ernsthaft krank bin.	○	○	○	○	○
Es macht mir Angst, wenn ich mich nicht auf eine Aufgabe konzentrieren kann.	○	○	○	○	○
Wenn ich in Gegenwart anderer zittere, fürchte ich, was diese Personen von mir denken.	○	○	○	○	○
Wenn ich ein Beklemmungsgefühl in der Brust habe, befürchte ich, dass ich nicht mehr richtig atmen kann.	○	○	○	○	○
Wenn ich Schmerzen in meiner Brust habe, befürchte ich, einen Herzinfarkt zu bekommen.	○	○	○	○	○
Es macht mir Sorgen, dass andere Personen meine Angst bemerken könnten.	○	○	○	○	○
Wenn ich das Gefühl habe neben mir zu stehen, befürchte ich, dass ich seelisch krank bin.	○	○	○	○	○
Es macht mir Angst, wenn ich vor anderen Menschen erröte.	○	○	○	○	○
Wenn ich bemerke, dass mein Herz für einen Moment aussetzt, befürchte ich, dass etwas mit mir nicht stimmt.	○	○	○	○	○
Wenn ich in Anwesenheit Anderer anfange zu schwitzen, fürchte ich, dass sie negativ über mich denken.	○	○	○	○	○
Wenn sich meine Gedanken beschleunigen, fürchte ich, dass ich verrückt werde.	○	○	○	○	○
Wenn sich meine Kehle eng anfühlt, habe ich Angst, dass ich ersticken könnte.	○	○	○	○	○
Wenn ich Schwierigkeiten habe, klar zu denken, befürchte ich, dass etwas mit mir nicht stimmt.	○	○	○	○	○
Ich glaube, dass es schrecklich für mich wäre, in der Öffentlichkeit in Ohnmacht zu fallen.	○	○	○	○	○
Wenn ich einen „Blackout“ habe, befürchte ich, dass mit mir etwas ganz und gar nicht stimmt.	○	○	○	○	○

Seite 17

29. Bitte beantworten Sie folgende Fragen:

	Nein	Selten	Manchmal	Oft	Meistens
Wenn Sie von einer Krankheit lesen oder hören, bekommen Sie dann Symptome ähnlich wie die der betreffenden Krankheit?	○	○	○	○	○
Wenn Sie eine körperliche Empfindung bemerken, ist es dann für Sie schwierig, an etwas anderes zu denken?	○	○	○	○	○
Wenn Sie eine körperliche Empfindung bemerken, machen Sie sich dann Sorgen darum?	○	○	○	○	○

30. Wie oft fühlten Sie sich im Verlauf der letzten 2 Wochen durch die folgenden Beschwerden beeinträchtigt?

	Überhaupt nicht	An einzelnen Tagen	An mehr als der Hälfte der Tage	Beinahe jeden Tag
Wenig Interesse oder Freude an Ihren Tätigkeiten	○	○	○	○
Niedergeschlagenheit, Schwermut oder Hoffnungslosigkeit	○	○	○	○

Seite 18

31. Was beschreibt am besten, wie Sie sich im Allgemeinen fühlen?

Geben Sie an, wie Sie sich **im Allgemeinen** fühlen. Es gibt keine richtigen und falschen Antworten. Überlegen Sie nicht lange.

	Fast nie	Manchmal	Oft	Fast immer
Ich bin vergnügt.	○	○	○	○
Ich fühle mich nervös und unruhig.	○	○	○	○
Ich bin mit mir selbst zufrieden.	○	○	○	○
Ich wünschte, ich könnte so glücklich sein, wie andere es scheinbar sind.	○	○	○	○
Ich fühle mich als Versager.	○	○	○	○
Ich fühle mich ausgeruht.	○	○	○	○
Ich bin ruhig und gelassen.	○	○	○	○
Ich glaube, dass mir meine Schwierigkeiten über den Kopf wachsen.	○	○	○	○
Ich mache mir zuviel Gedanken über unwichtige Dinge.	○	○	○	○
Ich bin glücklich.	○	○	○	○
Ich habe beunruhigende Gedanken.	○	○	○	○
Mir fehlt es an Selbstvertrauen.	○	○	○	○
Ich fühle mich geborgen.	○	○	○	○
Entscheidungen treffen fällt mir leicht	○	○	○	○
Ich fühle mich unzulänglich.	○	○	○	○
Ich bin zufrieden.	○	○	○	○
Unwichtige Gedanken gehen mir durch den Kopf und bedrücken mich.	○	○	○	○
Enttäuschungen nehme ich so schwer dass ich sie nicht vergessen kann.	○	○	○	○
Ich bin ausgeglichen.	○	○	○	○
Ich werde nervös und unruhig, wenn ich an meine derzeitigen Angelegenheiten denke.	○	○	○	○

Seite 19

32. Dürfen wir Sie für eine Folgebefragung kontaktieren?

Wir würden Sie gerne in ca. 6 Monaten nochmals kontaktieren, um Fragen zu stellen, die an diesen Fragebogen anknüpfen. Damit können wir lernen, welche langfristigen Auswirkungen eine solche Pandemie hat und wie man darauf reagieren kann. Auch wenn Sie jetzt zustimmen, können Sie später noch frei entscheiden, ob Sie tatsächlich teilnehmen möchten oder nicht.

Um Ihre Anonymität sicher zu stellen, wird Ihre E-Mail-Adresse getrennt von allen anderen erhobenen Daten abgespeichert, sie kann nicht mit Ihren Fragebogenantworten in Verbindung gebracht werden.

Falls wir Sie nicht kontaktieren sollen, klicken Sie auf Weiter.

- [ ] Ja, Sie dürfen mich für diese Folgeerhebung unter folgender E-Mail-Adresse kontaktieren:

Seite 20

33. Sie haben alle Fragen beantwortet, vielen Dank für Ihre Mühe!
Hier noch eine letzte Frage:

- ○ Ich habe die Fragen sinnvoll beantwortet, meine Angaben können für die wissenschaftliche Auswertung verwendet werden.
- ○ Ich wollte „nur mal gucken“, meine Angaben sollten besser nicht in eine wissenschaftliche Auswertung eingehen.

Letzte Seite

Vielen Dank für Ihre Teilnahme!

Informationen zum Coronavirus können Sie derzeit von vielen Stellen abrufen und auf vielen Seiten nachschlagen. Auf den folgenden Seiten finden Sie die Informationen zum Coronavirus, die von der Universität Würzburg und vom Universitätsklinikum Würzburg zusammengestellt worden sind; die Zusammenstellung erhebt keinen Anspruch auf Vollständigkeit. Als weitere Seite ist die der Bundesregierung angegeben.

- Universität Würzburg
- Universitätsklinikum Würzburg
- Bundesregierung

Sollten die von uns gestellten Fragen bei Ihnen negative Gefühle ausgelöst haben, die stärker als normalerweise sind und Ihre Funktionalität einschränken, zögern Sie bitte nicht, nach professioneller Hilfe zu fragen.

Sie können sich bei psychischer Belastung, unter anderen, an folgende Anlaufstellen wenden:

Wenn Sie in Würzburg sind:

- Krisendienst Würzburg, telefonisch erreichbar unter +49 (0) 931 571717
- Hochschulambulanz für Psychotherapie der Universität Würzburg
- Psychotherapeutische Beratungsstelle für Studierende der Universität Würzburg

Deutschlandweit:

- Psychologische Hilfe während der Corona-Krise
- Telefonseelsorge, telefonisch erreichbar unter +49 (0) 800 111 0 111, +49 (0) 800 111 0 222
- Nummer gegen Kummer, telefonisch erreichbar unter +49 (0) 800 111 0 550 (Erwachsene) oder +49 (0) 800 111 0 333 (Kinder)

International:

- Worldwide Crisis Hotlines

Alles Gute wünscht Ihnen
das Forschungsteam des Lehrstuhls für Psychologie I und des Zentrums für Psychische Gesundheit der Universität Würzburg.

Ihre Antworten wurden gespeichert, Sie können das Browser-Fenster nun schließen.

Studienverantwortlicher: Prof. Dr. Paul Pauli
Kontakt zur Studienleitung
Julius-Maximilians-Universität Würzburg
Datenschutzbeauftragter der Universität Würzburg
