# Supplementary material for: German college students’ mental health state and their willingness to use mental health prevention: An online survey during the COVID-19 pandemic
Source: Heliyon. 2025 Jan 31;11(3):e42290. doi: 10.1016/j.heliyon.2025.e42290 (PMC11849601; doi:10.1016/j.heliyon.2025.e42290)
Supplement: Multimedia component 3 [file mmc3.pdf]

cnb → base 04.10.2023, 14:21

B015

Willkommen zum Online-Fragebogen über emotionale Belastungen infolge der Coronavirus (COVID-19) Pandemie

Diese Studie wird vom Lehrstuhl für Psychologie I und dem Zentrum für Psychische Gesundheit der Universität Würzburg durchgeführt.

Ziel der Untersuchung

Sie wurden angeschrieben, da Sie bereits am ersten Teil dieser Studie im Frühjahr 2020 teilgenommen haben. Mit dieser Befragung wollen wir untersuchen, wie sich verschiedene psychologische Parameter mittlerweile entwickelt haben. Ziel dieser Untersuchung ist es, ein Verständnis dafür zu entwickeln, wie Individuen emotional auf die Coronavirus (COVID-19) Pandemie reagieren. Zu diesem Zweck möchten wir Sie darum bitten, einige Fragen zu aktuellen Sorgen und Ängsten, sozialer Unterstützung, sowie Ihrer momentanen beruflichen und persönlichen Lebenssituation zu beantworten.

Bearbeitungsdauer

Die Bearbeitung des kompletten Fragebogens wird **circa 30 Minuten** in Anspruch nehmen.

Freiwilligkeit

Die Teilnahme ist freiwillig. Sie können die Teilnahme jederzeit beenden, ohne dass Ihnen daraus Nachteile entstehen. Schließen Sie in diesem Fall einfach das Browserfenster, Ihre Daten werden dann als unvollständig markiert und zum Ende der Befragung gelöscht.

Kompensation

Zum Dank für eine Teilnahme verlosen wir unter allen Teilnehmenden, die den Fragebogen vollständig ausfüllen, mehrfach den Betrag von 50 €, sodass jede oder jeder 20-te Teilnehmende gewinnt. Die Teilnahme wird nicht anderweitig vergütet, außer über die Gewinnchance im Gewinnspiel.

Wenn Sie am Gewinnspiel teilnehmen möchten, hinterlassen Sie uns Ihre E-Mail-Adresse, damit wir Sie im Gewinnfall informieren können. Die E-Mail-Adresse wird nicht an Dritte weitergegeben und wird separat von Ihren Umfragedaten gespeichert, sodass alle Umfragedaten anonym bleiben. Die E-Mail-Adresse wird ausschließlich zu diesem Zweck gespeichert und spätestens am 31.03.2022 vollständig gelöscht. Zum Zweck der Auszahlung des Gewinns müssen persönliche Daten erhoben werden, die an das Servicezentrum für Finanzen der Universität Würzburg weitergeleitet werden müssen. Diese Daten beinhalten die IBAN, die vollständige Adresse (Straße, Hausnummer, PLZ, Ort), den Vornamen und den Nachnamen. Diese Daten werden nicht mit dem wissenschaftlichen Datensatz (Umfragedaten) zusammengeführt und werden nur zu Zwecken der Auszahlung der Kompensationsmittel der Versuchsteilnahme erhoben sowie an das Servicezentrum für Finanzen der Universität Würzburg weitergeleitet. Bei einer Überschreitung eines jährlichen Betrages von 1500 € durch Versuchsteilnahmen ist die Universität verpflichtet diese Einkünfte an das Finanzamt weiterzuleiten. Andernfalls erfolgt keine Weiterleitung.

Pseudonymisierung

Die Erhebung der Daten erfolgt pseudonymisiert. Mithilfe des Pseudonyms können wir die bereits erfolgte und die jetzige Erhebung zusammenfügen. Auf diese Weise können wir Veränderungen in Ihren Antworten nachvollziehen, ohne Ihre Identität zu kennen. Alle persönlichen Daten wie z.B. Name und Adresse werden streng getrennt von den Umfragedaten aufbewahrt und den Wissenschaftlern, die die wissenschaftlichen Analysen ausführen, nicht offengelegt. Ein direkter Rückgriff auf Ihre Person ist somit ausgeschlossen. Mit Hilfe Ihres Pseudonyms ist es möglich, die von Ihnen gemachten Angaben auch nachträglich noch zu löschen. Bitte bewahren sie es daher gut auf. Um Ihre Teilnahme zu widerrufen und/oder die Löschung Ihrer Daten in Teilen oder insgesamt zu veranlassen, nehmen Sie bitte Kontakt zur Studienleitung auf.

Datenschutz

Die pseudonymisiert erfassten Daten werden wissenschaftlich ausgewertet, in Gruppen zusammengefasst veröffentlicht und für einen Zeitraum von mindestens 10 Jahren in Übereinstimmung mit der Datenschutz-Grundverordnung der EU auf sicheren Servern aufbewahrt. Zudem können sie in vollständig anonymisierter Form über die Internet-Datenbank Open Science Framework zur Sicherstellung guter wissenschaftlicher Arbeit öffentlich zugänglich gemacht werden. Bitte beachten Sie hierzu auch die Informationen zur Verarbeitung personenbezogener Daten sowie zur DSGVO, die Sie über die Seite des Datenschutzbeauftragten der Universität Würzburg einsehen können.

Löschung der Daten

Im Rahmen der Befragung wird ein persönliches Codewort erstellt. Das Codewort erlaubt keine Rückschlüsse darauf, wer Sie sind. Mit diesem Codewort können Sie allerdings jederzeit die Löschung Ihrer Daten während des Aufbewahrungszeitraums verlangen, wenn Sie sich per Email an die Studienleitung wenden.
Mithilfe Ihres Codewortes können wir zudem Ihre Daten aus dieser Erhebung mit Ihren Daten aus der ersten Erhebung zusammenfügen, um so Veränderungen in Ihren Antworten nachvollziehen zu können, ohne Ihre Identität zu kennen.

Einverständniserklärung

B001

Sind Sie mit diesen Bedingungen einverstanden und möchten an der Studie teilnehmen?

Falls Sie nicht teilnehmen möchten, schließen Sie jetzt bitte das Browser-Fenster.

- ◯ Ja, ich bin einverstanden und möchte teilnehmen.

Seite 02

B002

Erstellung eines Pseudonyms

Mit Ihren Angaben auf dieser Seite wird ein Codewort (Pseudonym) erstellt. Sie können nach Beendigung der Studie die Löschung Ihrer Daten unter Angeben des Codeworts (z.B. raul13091990) verlangen. Außerdem können wir mithilfe Ihres Codewortes Ihre Daten aus dieser Erhebung mit Ihren Daten aus der ersten Erhebung zusammenfügen. **Bitte achten Sie daher darauf, den Anweisungen zur Erstellung des Codewortes genaustens Folge zu leisten.**

Wie lauten die beiden letzten Buchstaben des (ersten) Vornamens Ihrer Mutter?

Beispiel: **ra** bei Pet**ra** Anna Schmidt. **xx** falls unbekannt oder falls Sie keine Angabe machen möchten.

Wie lauten die beiden letzten Buchstaben des (ersten) Vornamens Ihres Vaters?

Beispiel: **ul** bei Pa**ul** Peter Schmidt. **xx** falls unbekannt oder falls Sie keine Angabe machen möchten.

Was ist Ihr Geburtsdatum?

B013

Welchem Geschlecht fühlen Sie sich zugehörig?

- ◯ männlich
- ◯ weiblich
- ◯ divers
- ◯ keine Angabe

B004

Was ist Ihre Staatsbürgerschaft?

Bei Mehrstaatigkeit bitte die Staatsbürgerschaft angeben, in dessen Staat Sie im vergangenen Jahr die meiste Zeit verbracht haben.

B016

In welchem Staat und Bundesland befanden Sie sich in den LETZTEN 6 MONATEN hauptsächlich?

Staat:

Bundesland:

B034

Mein Lebensmittelpunkt in den LETZTEN 6 MONATEN befand sich...

Bitte geben Sie an, was eher auf Ihren Wohnort zutrifft

- ◯ auf dem Land
- ◯ in einer Kleinstadt (> 5000 Einwohner)
- ◯ in einer Großstadt (> 100 000 Einwohner)

B017

Wer hat in den LETZTEN 6 MONATEN mit Ihnen im Haushalt gelebt?

(Mehrfachauswahl möglich)

- ☐ (Ehe-)Partner*in
- ☐ Kinder, 0 bis 6-jährig
- ☐ Kinder, 7 bis 17-jährig
- ☐ Kinder, 18-jährig und älter
- ☐ Eltern und/oder Großeltern
- ☐ WG-Mitbewohner/Geschwister
- ☐ Haustiere
- ☐ Sonstige

- ☐ Nur ich

B035

Meine Wohnsituation hat sich in den LETZTEN 6 MONATEN maßgeblich verändert?

- ◯ Ja
- ◯ Nein

B036

	Überhaupt nicht					Sehr
Wie zufrieden sind Sie mit Ihrer Wohnsituation in den **LETZTEN 6 MONATEN** ?	◯	◯	◯	◯	◯	◯

B025

Bis zu welchem Ausmaß waren oder sind Menschen in Ihrem Umfeld am Coronavirus erkrankt?

	Leichter Verlauf	Schwerer Verlauf	Long-Covid (längerfristige Einschränkungen)	Todesfall	Nicht betroffen
Im privaten Umfeld (Familie oder enge Freunde/Bekannte)	☐	☐	☐	☐	☐
Im beruflichen/studentischen Umfeld	☐	☐	☐	☐	☐
Im weiteren Bekanntenkreis	☐	☐	☐	☐	☐
Sie selbst	☐	☐	☐	☐	☐

B037

Welche Aussage trifft am ehesten auf Sie zu?

- ◯ Ich bin vollständig gegen COVID-19 geimpft (Erst- und ggf. Zweitimpfung).
- ◯ Ich bin teilweise gegen COVID-19 geimpft (nur Erstimpfung bei einem Impfstoff, der eine Zweitimpfung erfordert).
- ◯ Ich habe vor, mich demnächst gegen COVID-19 impfen zu lassen.
- ◯ Ich möchte mich aktuell nicht gegen COVID-19 impfen lassen.
- ◯ Ich kann mich aktuell nicht gegen COVID-19 impfen lassen.

Seite 05

B028

Wie wird bzw. hat die Coronavirus (COVID-19) Pandemie Sie in den LETZTEN 6 MONATEN in den folgenden Bereichen beeinflusst?

Antworten Sie nach Ihrem Gefühl, ohne lange nachzudenken.

Körperliche Leistungsfähigkeit	Sehr negativ	Negativ	Neutral	Positiv	Sehr positiv
Geistige/intellektuelle Leistungsfähigkeit	Sehr negativ	Negativ	Neutral	Positiv	Sehr positiv
Psychische/emotionale Befindlichkeit	Sehr negativ	Negativ	Neutral	Positiv	Sehr positiv
Schlaf	Sehr negativ	Negativ	Neutral	Positiv	Sehr positiv
Soziale Kontakte	Sehr negativ	Negativ	Neutral	Positiv	Sehr positiv
Finanzielle Situation	Sehr negativ	Negativ	Neutral	Positiv	Sehr positiv
Mobilität	Sehr negativ	Negativ	Neutral	Positiv	Sehr positiv
Zukunftspläne	Sehr negativ	Negativ	Neutral	Positiv	Sehr positiv

B032

Inwieweit treffen die folgenden Aussagen auf Ihren Tagesablauf der LETZTEN WOCHE zu?

	Trifft überhaupt nicht zu				Trifft völlig zu
Ich hatte eine klare Tagesstruktur.	○	○	○	○	○
Ich hatte angenehme Aktivitäten.	○	○	○	○	○
Ich hatte unangenehme Aktivitäten.	○	○	○	○	○
Ich war körperlich aktiv.	○	○	○	○	○
Ich hatte berufliche oder studentische Aufgaben.	○	○	○	○	○
Ich betreute Kinder (nicht von Berufs wegen).	○	○	○	○	○
Ich hatte sonstige Pflichten.	○	○	○	○	○
Ich vermied alle unnötigen Aktivitäten.	○	○	○	○	○

B019

Welche Tätigkeit üben Sie aktuell aus?

(Mehrfachauswahl möglich)

- ☐ Studium
- ☐ Ausbildung (bitte angeben): ________
- ☐ Erwerbstätigkeit, auch Teilzeit oder Nebenjob (bitte angeben): ________
- ☐ Arbeitslos/Arbeitssuchend
- ☐ Sonstiges (bitte angeben): ________

2 aktive(r) Filter

Filter B019/F1
Wenn eine der folgenden Antwortoption(en) ausgewählt wurde: **6**

Dann Seite(n) **PRpre** des Fragebogens anzeigen (sonst ausblenden)

Filter B019/F2
Wenn die Antwortoption(en) **6** nicht (alle) ausgewählt wurde(n).
Dann Seite(n) **PR** des Fragebogens ausblenden

B038

	Sehr negativ			Neutral			Sehr positiv
Meiner Empfindung nach hat sich meine Arbeitssituation aufgrund der Coronavirus (COVID-19) Pandemie wie folgt verändert:	○	○	○	○	○	○	○

Seite 06

B009

Wie oft haben Sie in den LETZTEN 2 WOCHEN nach Informationen zur Coronavirus (COVID-19) Pandemie geschaut?

- ◯ Nie
- ◯ Alle paar Tage
- ◯ Ein Mal pro Tag
- ◯ Mehrmals pro Tag
- ◯ Ein Mal pro Stunde
- ◯ Mehrmals pro Stunde

B011

Beantworten Sie die folgenden Fragen nach Ihrem Gefühl, ohne lange nachzudenken.

	Sehr wenig				Sehr stark
Wie wahrscheinlich ist es, dass Sie mit dem Coronavirus infiziert werden?	○	○	○	○	○
Wie wahrscheinlich ist es, dass jemand, den Sie kennen, mit dem Coronavirus infiziert wird?	○	○	○	○	○
Inwiefern zählen Sie sich zur Risikogruppe bei einer Coronavirus-Infektion (durch eigenes erhöhtes Risiko oder durch engen Kontakt zu gefährdeten Personen)?	○	○	○	○	○
Wie stark sind Sie mit Informationen über das Coronavirus konfrontiert?	○	○	○	○	○
Inwieweit sind Sie besorgt, bei einer Infektion mit dem Coronavirus schwer zu erkranken?	○	○	○	○	○
Inwieweit sind Sie besorgt, dass jemand, den Sie kennen, durch das Coronavirus schwer erkranken könnte?	○	○	○	○	○
Inwieweit hat die Bedrohung durch das Coronavirus Ihre Entscheidung beeinflusst, unter Leuten zu sein?	○	○	○	○	○
Inwieweit hat die Bedrohung durch das Coronavirus Ihre Reisepläne beeinflusst?	○	○	○	○	○
Inwieweit hat die Bedrohung durch das Coronavirus Ihre Anwendung von Sicherheitsverhalten (z.B. Handdesinfektion) beeinflusst?	○	○	○	○	○
Machen Sie sich Sorgen, aufgrund der Coronavirus (COVID-19) Pandemie Ihren Arbeitsplatz zu verlieren bzw. Ihr Studium nicht erfolgreich abschließen zu können?	○	○	○	○	○
Machen Sie sich Sorgen, aufgrund der Coronavirus (COVID-19) Pandemie in finanzielle Nöte zu geraten?	○	○	○	○	○
Fühlen Sie sich den Herausforderungen durch die Coronavirus (COVID-19) Pandemie gewachsen?	○	○	○	○	○
Sehen Sie Forschung seit der Coronavirus (COVID-19) Pandemie als mehr relevant an?	○	○	○	○	○
Sehen Sie Gesundheitsberufe seit der Coronavirus (COVID-19) Pandemie als mehr relevant an?	○	○	○	○	○

B012

Wie sehr treffen folgende Aussagen auf Ihr Verhalten in den LETZTEN 2 WOCHEN zu?

	Trifft überhaupt nicht zu				Trifft völlig zu
Wenn ich in den Lebensmittelladen ging, kaufte ich mehr Essen ein als sonst.	○	○	○	○	○
Ich zog es vor, zu Hause zu bleiben, um soziale Kontakte zu vermeiden.	○	○	○	○	○
Ich vermied Körperkontakt (z.B. Händeschütteln, Umarmungen).	○	○	○	○	○
Ich versuchte, das Berühren von potentiell kontaminierten Objekten zu vermeiden (z.B. Türklinken, Fahrstuhlknöpfe etc.).	○	○	○	○	○
Ich vermied soziale Aktivitäten wie Besuche von Restaurants, Theater, Kinos, etc.	○	○	○	○	○
Ich versuchte, einen Sicherheitsabstand zu Anderen einzuhalten.	○	○	○	○	○

B021

Wie sehr treffen folgende Aussagen aktuell auf Sie zu?

	Trifft überhaupt nicht zu				Trifft völlig zu
Ich fühle mich durch mein soziales Umfeld (Familie, Freunde) unterstützt.	○	○	○	○	○
Ich habe Angst, aufgrund der Corona-Krise zu vereinsamen.	○	○	○	○	○
Es macht mir nichts aus, öfter als sonst zu Hause zu sein.	○	○	○	○	○
Positive Interaktionen mit anwesenden Personen entspannen mich.	○	○	○	○	○
Digitale Interaktionen können persönliche Interaktionen ersetzen.	○	○	○	○	○
Mein Mitgefühl gilt ausschließlich den Corona-Opfern in meinem Heimatland.	○	○	○	○	○
Die Corona-Krise hat meine Einstellung gegenüber anderen Nationen negativ beeinflusst.	○	○	○	○	○

B039

Bitte geben Sie an, inwiefern die folgenden Aussagen über den LETZEN MONAT hinweg mit Ihnen übereinstimmen.

Wenn eine bestimmte Situation in letzter Zeit nicht aufgetreten ist, antworten Sie so, wie Sie gefühlt hätten, wenn die Situation aufgetreten wäre.

	Überhaupt nicht wahr	Selten wahr	Manchmal wahr	Oft wahr	Fast immer wahr
Ich bin fähig mich anzupassen, wenn sich etwas verändert.	○	○	○	○	○
Ich komme mit allem klar, was sich mir in den Weg stellt.	○	○	○	○	○
Wenn ich mit Problemen konfrontiert bin, versuche ich dies mit Humor zu sehen.	○	○	○	○	○
Der Umgang mit Stress kann mich stärken.	○	○	○	○	○
Ich neige dazu, mich nach Krankheit, Verletzungen oder anderen Missgeschicken wieder gut zu erholen.	○	○	○	○	○
Auch wenn es Hindernisse gibt, bin ich der Meinung meine Ziele erreichen zu können.	○	○	○	○	○
Wenn ich unter Druck stehe, bleibe ich fokussiert und denke klar.	○	○	○	○	○
Wenn ich versage, lasse ich mich nicht leicht entmutigen.	○	○	○	○	○
Wenn es um den Umgang mit Herausforderungen des Lebens und allgemeine Schwierigkeiten geht, schätze ich mich als starke Person ein.	○	○	○	○	○
Ich bin fähig mit unerfreulichen oder schmerzhaften Gefühlen wie Traurigkeit, Angst und Wut umzugehen.	○	○	○	○	○

B040

Wie stark fühlten Sie sich im Verlauf der LETZTEN 7 TAGE durch die folgenden Beschwerden beeinträchtigt?

	Gar nicht				Stark
Bauchschmerzen oder Verdauungsbeschwerden	○	○	○	○	○
Rückenschmerzen	○	○	○	○	○
Schmerzen in Armen, Beinen oder Gelenken	○	○	○	○	○
Kopfschmerzen	○	○	○	○	○
Schmerzen im Brustbereich oder Kurzatmigkeit	○	○	○	○	○
Schwindel	○	○	○	○	○
Müdigkeit oder Gefühl, keine Energie zu haben	○	○	○	○	○
Schlafstörungen	○	○	○	○	○

FC01

Wie sehr stimmen Sie folgenden Aussagen zu?

	Stimme überhaupt nicht zu	Stimme nicht zu	Weder noch	Stimme zu	Stimme völlig zu
Ich habe Angst vor dem Coronavirus-19.	○	○	○	○	○
Es ist mir unangenehm, über das Coronavirus-19 nachzudenken.	○	○	○	○	○
Meine Hände werden klamm, wenn ich über das Coronavirus-19 nachdenke.	○	○	○	○	○
Ich habe Angst davor, wegen des Coronavirus-19 mein Leben zu verlieren.	○	○	○	○	○
Ich werde ängstlich und nervös, wenn ich Nachrichten über das Coronavirus-19 auf sozialen Medien sehe.	○	○	○	○	○
Ich kann nicht schlafen, weil ich mir Sorgen darüber mache, das Coronavirus-19 zu bekommen.	○	○	○	○	○
Mein Herz rast oder pocht, wenn ich darüber nachdenke, das Coronavirus-19 zu bekommen.	○	○	○	○	○

PH01

Wie oft fühlten Sie sich im Verlauf der LETZTEN 2 WOCHEN durch die folgenden Beschwerden beeinträchtigt?

Wenig Interesse oder Freude an Ihren Tätigkeiten	Überhaupt nicht	An einzelnen Tagen	An mehr als der Hälfte der Tage	Beinahe jeden Tag
Niedergeschlagenheit, Schwermut oder Hoffnungslosigkeit	Überhaupt nicht	An einzelnen Tagen	An mehr als der Hälfte der Tage	Beinahe jeden Tag

ST03

Wie fühlen Sie sich jetzt, IN DIESEM MOMENT?

Geben Sie an, wie Sie sich **jetzt, in diesem Moment** fühlen. Es gibt keine richtigen und falschen Antworten. Überlegen Sie nicht lange und wählen Sie die Antwort, die Ihren augenblicklichen Gefühlszustand am besten beschreibt.

Ich bin ruhig.	Überhaupt nicht	Ein wenig	Ziemlich	Sehr
Ich fühle mich angespannt.	Überhaupt nicht	Ein wenig	Ziemlich	Sehr
Ich bin aufgeregt.	Überhaupt nicht	Ein wenig	Ziemlich	Sehr
Ich bin entspannt.	Überhaupt nicht	Ein wenig	Ziemlich	Sehr
Ich bin zufrieden.	Überhaupt nicht	Ein wenig	Ziemlich	Sehr
Ich bin besorgt.	Überhaupt nicht	Ein wenig	Ziemlich	Sehr

IU02

Wie sehr treffen folgende Aussagen auf Sie zu?

Unvorhergesehene Ereignisse regen mich sehr auf.	Überhaupt nicht	Wenig	Teils teils	Sehr	Völlig
Es frustriert mich, wenn ich nicht alle Informationen habe, die ich brauche.	Überhaupt nicht	Wenig	Teils teils	Sehr	Völlig
Unsicherheit hält mich davon ab, ein erfülltes Leben zu führen.	Überhaupt nicht	Wenig	Teils teils	Sehr	Völlig
Man sollte immer nach vorne schauen, um Überraschungen zu vermeiden.	Überhaupt nicht	Wenig	Teils teils	Sehr	Völlig
Ein kleines unvorhergesehenes Ereignis kann alles verderben, selbst bei bester Planung.	Überhaupt nicht	Wenig	Teils teils	Sehr	Völlig
Wenn es Zeit zum Handeln ist, lähmt mich die Unsicherheit.	Überhaupt nicht	Wenig	Teils teils	Sehr	Völlig
Wenn ich unsicher bin, kann ich nicht sehr gut funktionieren.	Überhaupt nicht	Wenig	Teils teils	Sehr	Völlig
Ich möchte immer wissen, was die Zukunft für mich bereithält.	Überhaupt nicht	Wenig	Teils teils	Sehr	Völlig
Ich kann es nicht ertragen, wenn man mich überrascht.	Überhaupt nicht	Wenig	Teils teils	Sehr	Völlig
Der kleinste Zweifel kann mich vom Handeln abhalten.	Überhaupt nicht	Wenig	Teils teils	Sehr	Völlig
Ich sollte in der Lage sein, alles im Voraus zu organisieren.	Überhaupt nicht	Wenig	Teils teils	Sehr	Völlig
Ich muss aus allen unsicheren Situationen herauskommen.	Überhaupt nicht	Wenig	Teils teils	Sehr	Völlig

Seite 13

PS01

Wie typisch oder charakteristisch sind folgende Aussagen für Sie?

Wenn ich nicht genug Zeit habe, alles zu erledigen, mache ich mir darüber keine Sorgen.	Überhaupt nicht typisch	Nur wenig typisch	Ziemlich typisch	Sehr typisch	Äußerst typisch
Meine Sorgen wachsen mir über den Kopf.	Überhaupt nicht typisch	Nur wenig typisch	Ziemlich typisch	Sehr typisch	Äußerst typisch
Ich neige nicht dazu, mir über Dinge Sorgen zu machen.	Überhaupt nicht typisch	Nur wenig typisch	Ziemlich typisch	Sehr typisch	Äußerst typisch
Viele Situationen machen mir Sorgen.	Überhaupt nicht typisch	Nur wenig typisch	Ziemlich typisch	Sehr typisch	Äußerst typisch
Ich weiß, ich sollte mir keine Sorgen machen, aber ich kann nichts dagegen machen.	Überhaupt nicht typisch	Nur wenig typisch	Ziemlich typisch	Sehr typisch	Äußerst typisch
Wenn ich unter Druck stehe, mache ich mir viel Sorgen.	Überhaupt nicht typisch	Nur wenig typisch	Ziemlich typisch	Sehr typisch	Äußerst typisch
Über irgendetwas mache ich mir immer Sorgen.	Überhaupt nicht typisch	Nur wenig typisch	Ziemlich typisch	Sehr typisch	Äußerst typisch
Mir fällt es leicht, sorgenvolle Gedanken zu vertreiben.	Überhaupt nicht typisch	Nur wenig typisch	Ziemlich typisch	Sehr typisch	Äußerst typisch
Sobald ich eine Aufgabe beendet habe, fange ich an, mir über all das Sorgen zu machen, was ich sonst noch tun muss.	Überhaupt nicht typisch	Nur wenig typisch	Ziemlich typisch	Sehr typisch	Äußerst typisch
Ich mache mir nie über etwas Sorgen.	Überhaupt nicht typisch	Nur wenig typisch	Ziemlich typisch	Sehr typisch	Äußerst typisch
Wenn ich in einer Angelegenheit nichts mehr tun kann, mache ich mir auch keine Sorgen mehr darüber.	Überhaupt nicht typisch	Nur wenig typisch	Ziemlich typisch	Sehr typisch	Äußerst typisch

Ich war schon immer jemand, der sich viel Sorgen macht.	Überhaupt nicht typisch	Nur wenig typisch	Ziemlich typisch	Sehr typisch	Äußerst typisch
Mir fällt auf, dass ich mir über einiges Sorgen gemacht habe.	Überhaupt nicht typisch	Nur wenig typisch	Ziemlich typisch	Sehr typisch	Äußerst typisch
Wenn ich erst einmal anfange, mir Sorgen zu machen, kann ich nicht mehr damit aufhören.	Überhaupt nicht typisch	Nur wenig typisch	Ziemlich typisch	Sehr typisch	Äußerst typisch
Ich mache mir die ganze Zeit über Sorgen.	Überhaupt nicht typisch	Nur wenig typisch	Ziemlich typisch	Sehr typisch	Äußerst typisch
Ich mache mir über Vorhaben solange Sorgen, bis sie komplett erledigt sind.	Überhaupt nicht typisch	Nur wenig typisch	Ziemlich typisch	Sehr typisch	Äußerst typisch

Seite 14

AS01

Zu welchem Grad stimmen Sie folgenden Aussagen zu?

Sollte eine Aussage Inhalte thematisieren, die Sie nicht erlebt haben, antworten Sie bitte gemäß der Erwartung, wie Sie sich bei einer solchen Erfahrung fühlen würden.

Es ist mir wichtig, nicht nervös zu erscheinen.	Sehr wenig	Wenig	Teils teils	Ziemlich	Sehr stark
Wenn ich mich nicht auf eine Aufgabe konzentrieren kann, befürchte ich, verrückt zu werden.	Sehr wenig	Wenig	Teils teils	Ziemlich	Sehr stark
Es macht mir Angst, wenn ich starkes Herzklopfen verspüre.	Sehr wenig	Wenig	Teils teils	Ziemlich	Sehr stark
Wenn ich mir den Magen verdorben habe, befürchte ich, dass ich ernsthaft krank bin.	Sehr wenig	Wenig	Teils teils	Ziemlich	Sehr stark
Es macht mir Angst, wenn ich mich nicht auf eine Aufgabe konzentrieren kann.	Sehr wenig	Wenig	Teils teils	Ziemlich	Sehr stark
Wenn ich in Gegenwart anderer zittere, fürchte ich, was diese Personen von mir denken.	Sehr wenig	Wenig	Teils teils	Ziemlich	Sehr stark
Wenn ich ein Beklemmungsgefühl in der Brust habe, befürchte ich, dass ich nicht mehr richtig atmen kann.	Sehr wenig	Wenig	Teils teils	Ziemlich	Sehr stark
Wenn ich Schmerzen in meiner Brust habe, befürchte ich, einen Herzinfarkt zu bekommen.	Sehr wenig	Wenig	Teils teils	Ziemlich	Sehr stark
Es macht mir Sorgen, dass andere Personen meine Angst bemerken könnten.	Sehr wenig	Wenig	Teils teils	Ziemlich	Sehr stark
Wenn ich das Gefühl habe neben mir zu stehen, befürchte ich, dass ich seelisch krank bin.	Sehr wenig	Wenig	Teils teils	Ziemlich	Sehr stark
Es macht mir Angst, wenn ich vor anderen Menschen erröte.	Sehr wenig	Wenig	Teils teils	Ziemlich	Sehr stark
Wenn ich bemerke, dass mein Herz für einen Moment aussetzt, befürchte ich, dass etwas mit mir nicht stimmt.	Sehr wenig	Wenig	Teils teils	Ziemlich	Sehr stark
Wenn ich in Anwesenheit Anderer anfange zu schwitzen, fürchte ich, dass sie negativ über mich denken.	Sehr wenig	Wenig	Teils teils	Ziemlich	Sehr stark

Wenn sich meine Gedanken beschleunigen, fürchte ich, dass ich verrückt werde.	Sehr wenig	Wenig	Teils teils	Ziemlich	Sehr stark
Wenn sich meine Kehle eng anfühlt, habe ich Angst, dass ich ersticken könnte.	Sehr wenig	Wenig	Teils teils	Ziemlich	Sehr stark
Wenn ich Schwierigkeiten habe, klar zu denken, befürchte ich, dass etwas mit mir nicht stimmt.	Sehr wenig	Wenig	Teils teils	Ziemlich	Sehr stark
Ich glaube, dass es schrecklich für mich wäre, in der Öffentlichkeit in Ohnmacht zu fallen.	Sehr wenig	Wenig	Teils teils	Ziemlich	Sehr stark
Wenn ich einen „Blackout“ habe, befürchte ich, dass mit mir etwas ganz und gar nicht stimmt.	Sehr wenig	Wenig	Teils teils	Ziemlich	Sehr stark

---

Seite 15

IA01

Bitte beantworten Sie folgende Fragen:

Wenn Sie von einer Krankheit lesen oder hören, bekommen Sie dann Symptome ähnlich wie die der betreffenden Krankheit?	Nein	Selten	Manchmal	Oft	Meistens
Wenn Sie eine körperliche Empfindung bemerken, ist es dann für Sie schwierig, an etwas anderes zu denken?	Nein	Selten	Manchmal	Oft	Meistens
Wenn Sie eine körperliche Empfindung bemerken, machen Sie sich dann Sorgen darum?	Nein	Selten	Manchmal	Oft	Meistens

Seite 16

ST02

Was beschreibt am besten, wie Sie sich IM ALLGEMEINEN fühlen?

Geben Sie an, wie Sie sich **im Allgemeinen** fühlen. Es gibt keine richtigen und falschen Antworten. Überlegen Sie nicht lange.

Ich bin vergnügt.	Fast nie	Manchmal	Oft	Fast immer
Ich fühle mich nervös und unruhig.	Fast nie	Manchmal	Oft	Fast immer
Ich bin mit mir selbst zufrieden.	Fast nie	Manchmal	Oft	Fast immer
Ich wünschte, ich könnte so glücklich sein, wie andere es scheinbar sind.	Fast nie	Manchmal	Oft	Fast immer
Ich fühle mich als Versager.	Fast nie	Manchmal	Oft	Fast immer
Ich fühle mich ausgeruht.	Fast nie	Manchmal	Oft	Fast immer
Ich bin ruhig und gelassen.	Fast nie	Manchmal	Oft	Fast immer
Ich glaube, dass mir meine Schwierigkeiten über den Kopf wachsen.	Fast nie	Manchmal	Oft	Fast immer
Ich mache mir zuviel Gedanken über unwichtige Dinge.	Fast nie	Manchmal	Oft	Fast immer
Ich bin glücklich.	Fast nie	Manchmal	Oft	Fast immer
Ich habe beunruhigende Gedanken.	Fast nie	Manchmal	Oft	Fast immer
Mir fehlt es an Selbstvertrauen.	Fast nie	Manchmal	Oft	Fast immer
Ich fühle mich geborgen.	Fast nie	Manchmal	Oft	Fast immer

Entscheidungen treffen fällt mir leicht.	Fast nie	Manchmal	Oft	Fast immer
Ich fühle mich unzulänglich.	Fast nie	Manchmal	Oft	Fast immer
Ich bin zufrieden.	Fast nie	Manchmal	Oft	Fast immer
Unwichtige Gedanken gehen mir durch den Kopf und bedrücken mich.	Fast nie	Manchmal	Oft	Fast immer
Enttäuschungen nehme ich so schwer, dass ich sie nicht vergessen kann.	Fast nie	Manchmal	Oft	Fast immer
Ich bin ausgeglichen.	Fast nie	Manchmal	Oft	Fast immer
Ich werde nervös und unruhig, wenn ich an meine derzeitigen Angelegenheiten denke.	Fast nie	Manchmal	Oft	Fast immer

Seite 17

AD01

Wie sehr beschreiben folgende Aussagen Ihr Befinden während der LETZTEN WOCHE?

Bitte kreuzen Sie bei folgenden Aussagen die Antwort an, die Ihrem Befinden während der letzten Woche am besten entspricht / entsprochen hat:

0: selten oder überhaupt nicht (weniger als 1 Tag)

1: manchmal (1 bis 2 Tage lang)

2: öfters (3 bis 4 Tage lang)

3: meistens, die ganze Zeit (5 bis 7 Tage lang)

Während der **LETZTEN WOCHE** ... haben mich Dinge beunruhigt, die mir sonst nichts ausmachen.	selten (0)	manchmal (1)	öfters (2)	meistens (3)
Während der **LETZTEN WOCHE** ... konnte ich meine trübsinnige Laune nicht loswerden, obwohl mich meine Freunde/Familie versuchten, aufzumuntern.	selten (0)	manchmal (1)	öfters (2)	meistens (3)
Während der **LETZTEN WOCHE** ... hatte ich Mühe, mich zu konzentrieren.	selten (0)	manchmal (1)	öfters (2)	meistens (3)
Während der **LETZTEN WOCHE** ... war ich deprimiert/niedergeschlagen.	selten (0)	manchmal (1)	öfters (2)	meistens (3)
Während der **LETZTEN WOCHE** ... war alles anstrengend für mich.	selten (0)	manchmal (1)	öfters (2)	meistens (3)
Während der **LETZTEN WOCHE** ... dachte ich, mein Leben ist ein einziger Fehlschlag.	selten (0)	manchmal (1)	öfters (2)	meistens (3)
Während der **LETZTEN WOCHE** ... hatte ich Angst.	selten (0)	manchmal (1)	öfters (2)	meistens (3)
Während der **LETZTEN WOCHE** ... habe ich schlecht geschlafen.	selten (0)	manchmal (1)	öfters (2)	meistens (3)
Während der **LETZTEN WOCHE** ... war ich fröhlich gestimmt.	selten (0)	manchmal (1)	öfters (2)	meistens (3)
Während der **LETZTEN WOCHE** ... habe ich weniger als sonst geredet.	selten (0)	manchmal (1)	öfters (2)	meistens (3)
Während der **LETZTEN WOCHE** ... fühlte ich mich einsam.	selten (0)	manchmal (1)	öfters (2)	meistens (3)

Während der **LETZTEN WOCHE** ... habe ich das Leben genossen.	selten (0)	manchmal (1)	öfters (2)	meistens (3)
Während der **LETZTEN WOCHE** ... war ich traurig.	selten (0)	manchmal (1)	öfters (2)	meistens (3)
Während der **LETZTEN WOCHE** ... hatte ich das Gefühl, dass mich die Leute nicht leiden können.	selten (0)	manchmal (1)	öfters (2)	meistens (3)
Während der **LETZTEN WOCHE** ... konnte ich mich zu nichts aufraffen.	selten (0)	manchmal (1)	öfters (2)	meistens (3)

SD02

Wichtige Anmerkung:

Die folgenden Aussagen beziehen sich auf ihr **aktuelles** Erleben und Empfinden. Bitte geben Sie an, in welchem Ausmaß jede der Aussagen Sie **aktuell** beschreibt.

SD01

1. Bitte geben Sie an, in welchem Ausmaß jede der folgenden Aussagen Sie *aktuell* beschreibt.

	aktuelles Erleben und Empfinden				
	stimmt völlig	stimmt ziemlich	teilsteils	stimmt wenig	stimmt gar nicht
Ich bin unzufrieden darüber, dass ich so zurückgezogen sein muss.					
Ich habe Menschen, mit denen ich sprechen kann.					
Ich fühle mich allein.					
Ich vermisse den persönlichen Kontakt zu Menschen, die zur Infektions-Risikogruppe (COVID-19) gehören.					
Ich fühle mich ausgeschlossen.					
Ich bin unglücklich darüber, dass ich so viele Dinge allein tun muss.					
Ich warte darauf, dass die Leute anrufen oder mir schreiben.					
Es gibt eine besondere Person, mit der ich meine Freuden und Sorgen teilen kann.					

Ich habe das Gefühl, dass sich meine Beziehung zu Freunden verschlechtert hat.

aktuelles Erleben und Empfinden

stimmt völlig	stimmt ziemlich	teilsteils	stimmt wenig	stimmt gar nicht

Ich bin von den Anderen isoliert.

aktuelles Erleben und Empfinden

stimmt völlig	stimmt ziemlich	teilsteils	stimmt wenig	stimmt gar nicht

Mir fehlt die Gesellschaft Anderer.

aktuelles Erleben und Empfinden

stimmt völlig	stimmt ziemlich	teilsteils	stimmt wenig	stimmt gar nicht

Ich bin zu viel allein.

aktuelles Erleben und Empfinden

stimmt völlig	stimmt ziemlich	teilsteils	stimmt wenig	stimmt gar nicht

SD03

Wichtige Anmerkung:

Die folgenden Aussagen beziehen sich auf Ihr Erleben und Empfinden **im Allgemeinen**.

Die folgenden Aussagen beziehen sich also nicht (nur) auf Ihr aktuelles Erleben und Empfinden. Bitte beachten Sie diese wichtige Unterscheidung.

SD04

2. Bitte geben Sie an, in welchem Ausmaß jede der folgenden Aussagen Sie i*m Allgemeinen* (also nicht erst seit Beginn der Corona-Pandemie) beschreibt.

Erleben und Empfinden im Allgemeinen

	stimmt völlig	stimmt ziemlich	teils teils	stimmt wenig	stimmt gar nicht
Ich bin ein geselliger Mensch.					
Ich bin einsam.					
Ich fühle mich niemandem nah.					
Ich habe viel gemeinsam mit den Menschen um mich herum.					
Ich fühle mich wohl, wenn ich unter Menschen bin.					
Ich erhalte von meinen Freunden und/oder meiner Familie emotionale Hilfe und Unterstützung.					
Meine Freundschaften sind oberflächlich.					
Niemand kennt mich wirklich gut.					

Ich wünsche mir mehr Kontakt zu Menschen, mit denen ich reden kann.

Erleben und Empfinden im Allgemeinen

stimmt völlig	stimmt ziemlich	teils teils	stimmt wenig	stimmt gar nicht

Ich finde soziale Anlässe oft beunruhigend.

Erleben und Empfinden im Allgemeinen

stimmt völlig	stimmt ziemlich	teils teils	stimmt wenig	stimmt gar nicht

Es fällt mir leicht, mich mit anderen Menschen zu entspannen.

Erleben und Empfinden im Allgemeinen

stimmt völlig	stimmt ziemlich	teils teils	stimmt wenig	stimmt gar nicht

Ich fühle mich ausgeschlossen.

Erleben und Empfinden im Allgemeinen

stimmt völlig	stimmt ziemlich	teils teils	stimmt wenig	stimmt gar nicht

Ich bin von den anderen isoliert.

Erleben und Empfinden im Allgemeinen

stimmt völlig	stimmt ziemlich	teils teils	stimmt wenig	stimmt gar nicht

B043

Das Deutsche Zentrum für Präventionsforschung und Psychische Gesundheit möchte Ihnen zum Abschluss dieser Studie fünf Fragen zur Verbesserung von Präventionsprogrammen stellen. Möchten Sie diese Fragen beantworten?

Ihre Gewinnchance ist davon unberührt.

- ◯ Ja
- ◯ Nein

1 aktive(r) Filter

Filter B043/F1
Wenn eine der folgenden Antwortoption(en) ausgewählt wurde: **1**
Dann Seite(n) **PR** des Fragebogens anzeigen (sonst ausblenden)

B044

Fragen des Deutschen Zentrums für Präventionsforschung und Psychische Gesundheit (DZPP)

Im Jahr 2019 wurde das Deutsche Zentrum für Präventionsforschung und Psychische Gesundheit (DZPP) in Kooperation zwischen Universität und Universitätsklinikum Würzburg gegründet. Das DZPP verfolgt das übergeordnete Ziel, innovative Präventionsprogramme zur Verbesserung der psychischen Gesundheit zu entwickeln, zu evaluieren und in der Fläche verfügbar zu machen. Für eine zielgruppenorientierte Entwicklung solcher Programme ist es in einem ersten Schritt notwendig den Bedarf nach Präventions- bzw. Unterstützungsangeboten der Studierenden zu eruieren. Bei den folgenden Fragen geht es daher vorerst um eine Bedarfserfassung auf deren Grundlage sich im Verlauf Angebote für eine settingbasierte Prävention entwickeln könnten.

PR01

	Gar nicht						Sehr
Wie groß ist Ihr generelles Interesse an mehr Informationen rund um das Thema psychische Gesundheit?	○	○	○	○	○	○	○

PR02

Bitte geben Sie an, wie wahrscheinlich Ihre Nutzung oder Ihre Teilnahme an folgenden Angeboten zum Thema psychische Gesundheit wäre.

	Überhaupt nicht						Sehr wahrscheinlich
Informationsbroschüren	○	○	○	○	○	○	○
Online-Kurs mit 4-8 Einheiten (Dauer ca. 1,5 h pro Einheit)	○	○	○	○	○	○	○
Präsenz-Workshops mit 4-8 Einheiten (Dauer ca. 1,5 h pro Einheit)	○	○	○	○	○	○	○
Online-Kurs zu spezifischem Thema, Inhalt frei wählbar (Dauer ca. 3 h)	○	○	○	○	○	○	○
Präsenz-Workshops zu spezifischen Themen, Inhalt frei wählbar (Dauer ca. 3 h)	○	○	○	○	○	○	○
Mehrwöchiges Seminar über mehrere Themenbereiche (Dauer ca. 1,5 h pro Woche)	○	○	○	○	○	○	○

Welche Themenbereiche interessieren Sie vorwiegend? PR03

	Überhaupt nicht						Viel Interesse
Psychische Störungen (Überblick über Störungsbilder, Psychoedukation)	○	○	○	○	○	○	○
Selbstschädigende Verhaltensweisen (Alkohol-, Drogenkonsum, Selbstverletzung)	○	○	○	○	○	○	○
Emotionsregulation	○	○	○	○	○	○	○
Self-compassion	○	○	○	○	○	○	○
Mindfulness (Achtsamkeit)	○	○	○	○	○	○	○
Förderung von Resilienz (Stärkung von Ressourcen)	○	○	○	○	○	○	○
Training zum Umgang mit belasteten Kommiliton*innen	○	○	○	○	○	○	○

PR04

Kennen Sie entsprechende Kontaktstellen an die Sie sich wenden könnten bei Bedarf? (während einer psychischen Krise von sich selbst oder Bekannten)

○ Ja, und zwar folgende: ______

○ Nein

PR05

Haben Sie während der Covid-19 Pandemie Maßnahmen zur Stärkung Ihrer psychischen Gesundheit bzw. zum Erhalt ihrer Lebensqualität ergriffen?

- ☐ (mehr) Sport (Joggen, Wandern, Yoga, etc.)
- ☐ Neues Hobby (Malen, Töpfern, andere Kunstformen)
- ☐ Kontakt zu Familie/ Freunden via Telefon/ Skype/ Facetime gesucht
- ☐ Strukturergreifende Maßnahmen (Kleiderschrank ordnen, Keller ausräumen, etc.)
- ☐ Handwerkliche Projekte (Hochbeet bauen, Tiere schnitzen, etc.)
- ☐ Haustier
- ☐ Andere

B041

Zum Dank für Ihre Teilnahme verlosen wir unter allen Teilnehmenden mehrfach den Betrag von 50 €. Jede oder jeder 20-te Teilnehmende gewinnt.

Wenn Sie am Gewinnspiel teilnehmen möchten, hinterlassen Sie uns Ihre E-Mail-Adresse, damit wir Sie im Gewinnfall informieren können. Die E-Mail-Adresse wird nicht an Dritte weitergegeben und wird separat von Ihren Umfragedaten gespeichert, sodass alle Umfragedaten anonym bleiben. Die E-Mail-Adresse wird ausschließlich zu diesem Zweck gespeichert und spätestens am 31.03.2022 vollständig gelöscht. Zum Zweck der Auszahlung des Gewinns müssen persönliche Daten erhoben werden, die an das Servicezentrum für Finanzen der Universität Würzburg weitergeleitet werden müssen. Diese Daten beinhalten die IBAN, die vollständige Adresse (Straße, Hausnummer, PLZ, Ort), den Vornamen und den Nachnamen. Diese Daten werden nicht mit dem wissenschaftlichen Datensatz (Umfragedaten) zusammengeführt und werden nur zu Zwecken der Auszahlung der Kompensationsmittel der Versuchsteilnahme erhoben sowie an das Servicezentrum für Finanzen der Universität Würzburg weitergeleitet. Bei einer Überschreitung eines jährlichen Betrages von 1500 € durch Versuchsteilnahmen ist die Universität verpflichtet diese Einkünfte an das Finanzamt weiterzuleiten. Andernfalls erfolgt keine Weiterleitung.

- [ ] Ja, ich möchte am Gewinnspiel teilnehmen und meine E-Mail-Adresse hierfür hinterlassen. Meine E-Mail-Adresse wird ausschließlich zu diesem Zwecke genutzt und spätestens am 31.03.2022 gelöscht.

B024

**Sie haben alle Fragen beantwortet, vielen Dank für Ihre Mühe!
Hier noch eine letzte Frage:**

- ◯ Ich habe die Fragen sinnvoll beantwortet, meine Angaben können für die wissenschaftliche Auswertung verwendet werden.
- ◯ Ich wollte „nur mal gucken“, meine Angaben sollten besser nicht in eine wissenschaftliche Auswertung eingehen.

B045

Dürfen wir Sie für Nachbefragungen zur Auswirkung der Corona-Pandemie per E-Mail kontaktieren? Sie helfen damit Erkenntnisse über die psychischen Auswirkungen der Pandemie zu erlangen.

Wenn Sie zustimmen, werden Ihre Kontaktdaten bis zum Abschluss der Studienreihe „emotionale Belastungen infolge der Coronavirus (COVID-19) Pandemie“ oder bis zum Widerruf Ihrer Einwilligung (per E-Mail an coronastudie@uni-wuerzburg.de) gespeichert und ausschließlich für die Kontaktaufnahme zu Folgebefragungen dieser Studienreihe verwendet. Eine Nichteinwilligung oder Widerruf ist mit keinerlei Nachteilen verbunden und führt zur unmittelbaren Löschung meiner Kontaktdaten.

Wenn Sie nicht zu stimmen, werden Ihre Kontaktdaten nach dieser Untersuchung gelöscht.

- ◯ Ja
- ◯ Nein

Letzte Seite

Vielen Dank für Ihre Teilnahme!

Sollten die von uns gestellten Fragen bei Ihnen negative Gefühle ausgelöst haben, die stärker als normalerweise sind und Ihre Funktionalität einschränken, zögern Sie bitte nicht, nach professioneller Hilfe zu fragen.

Sie können sich bei psychischer Belastung, unter anderem, an folgende Anlaufstellen wenden:

Wenn Sie in Würzburg sind:

- Krisendienst Würzburg, telefonisch erreichbar unter +49 (0) 931 571717
- Hochschulambulanz für Psychotherapie der Universität Würzburg
- Psychotherapeutische Beratungsstelle für Studierende der Universität Würzburg

Deutschlandweit:

- Psychologische Hilfe während der Corona-Krise
- Telefonseelsorge, telefonisch erreichbar unter +49 (0) 800 111 0 111, +49 (0) 800 111 0 222
- Nummer gegen Kummer, telefonisch erreichbar unter +49 (0) 800 111 0 550 (Erwachsene) oder +49 (0) 800 111 0 333 (Kinder)

International:

- Worldwide Crisis Hotlines

Alles Gute wünscht Ihnen
das Forschungsteam des Lehrstuhls für Psychologie I und des Zentrums für Psychische Gesundheit der Universität Würzburg.

Ihre Antworten wurden gespeichert, Sie können das Browser-Fenster nun schließen.

Studienverantwortliche: Prof. Dr. Matthias Gamer und Prof. Dr. Grit Hein
Kontakt zur Studienleitung
Julius-Maximilians-Universität Würzburg
Datenschutzbeauftragter der Universität Würzburg
